# Anisotropic dislocation-domain wall interactions in ferroelectrics

**DOI:** 10.1038/s41467-022-34304-7

**Published:** 2022-11-05

**Authors:** Fangping Zhuo, Xiandong Zhou, Shuang Gao, Marion Höfling, Felix Dietrich, Pedro B. Groszewicz, Lovro Fulanović, Patrick Breckner, Andreas Wohninsland, Bai-Xiang Xu, Hans-Joachim Kleebe, Xiaoli Tan, Jurij Koruza, Dragan Damjanovic, Jürgen Rödel

**Affiliations:** 1 Department of Materials and Earth Sciences, Technical University of Darmstadt, 64287 Darmstadt, Germany; 2 Key Laboratory of Advanced Technologies of Materials (Ministry of Education), School of Materials Science and Engineering, Southwest Jiaotong University, Chengdu, 610031 P. R. China; 3 Department of Physics, Technical University of Denmark, 2800 Kgs. Lyngby, Denmark; 4 Institute of Physical Chemistry, Technical University of Darmstadt, 64287 Darmstadt, Germany; 5 Department of Radiation Science and Technology, Delft University of Technology, Delft, 2629JB Netherlands; 6 Department of Materials Science and Engineering, Iowa State University, Ames, IA 50011 USA; 7 Institute for Chemistry and Technology of Materials, Graz University of Technology, A-8010 Graz, Austria; 8 Institute of Materials, École Polytechnique Fédérale de Lausanne, 1015 Lausanne, Switzerland

**Keywords:** Ferroelectrics and multiferroics, Mechanical engineering, Theory and computation

## Abstract

Dislocations are usually expected to degrade electrical, thermal and optical functionality and to tune mechanical properties of materials. Here, we demonstrate a general framework for the control of dislocation–domain wall interactions in ferroics, employing an imprinted dislocation network. Anisotropic dielectric and electromechanical properties are engineered in barium titanate crystals via well-controlled line-plane relationships, culminating in extraordinary and stable large-signal dielectric permittivity (≈23100) and piezoelectric coefficient (≈2470 pm V^–1^). In contrast, a related increase in properties utilizing point-plane relation prompts a dramatic cyclic degradation. Observed dielectric and piezoelectric properties are rationalized using transmission electron microscopy and time- and cycle-dependent nuclear magnetic resonance paired with X-ray diffraction. Succinct mechanistic understanding is provided by phase-field simulations and driving force calculations of the described dislocation–domain wall interactions. Our 1D-2D defect approach offers a fertile ground for tailoring functionality in a wide range of functional material systems.

## Abstract (web summary)

Dislocations are often perceived as a culprit for degradation in functionality. Here, the authors introduce a general framework for engineering dislocations and domain walls and demonstrate its full potential on a ferroelectric BaTiO_3_ single crystal.

## Introduction

Defects are ubiquitous in materials^1^, and defect engineering has become a key parameter of modern technology^2^, as for example in semiconductor and energy industries. Among them, topological one-dimensional (1D) dislocations and their associated elastic strain fields are of fundamental interest in materials science, with the control of dislocations emerging as a powerful approach for radically tailoring the electronic, thermal, and optical properties, such as superconductivity^3,4^, electrical/thermal conductivity^5,6^, and optical bandgap^7^. In particular during the last five years, extensive efforts have been made to significantly advance the knowledge about the interaction between dislocations and elementary particles^1,3–7^, such as phonons and electrons. Thanks to the planar strain field surrounded by dislocations, these are strongly scattered only when they propagate perpendicular to the dislocations^8^. This scenario offers guidance for altering intrinsically anisotropic functionalities, for example, dislocation-induced thermal transport anisotropy^9^ and enhanced superconductivity^4^.

Interestingly, when dislocations are interacting with defects of higher dimensionality such as domain walls known as topological two-dimensional (2D) defects^10^, the dislocations themselves act both as the sites for domain nucleation and as pinning centers for the motion of domain walls. For instance, dislocations can be used as a template for enabling nontrivial domain pinning/depinning phenomena^11,12^, and controlling the polarization instability in nanoscale ferroelectrics^13,14^. Recently, we addressed a strategy for imprinting dislocation networks with {101}<101> slip systems, using high-temperature creep compression along the [001] direction on BaTiO_3_ single crystals. The local dislocation–domain wall (DDW) interaction and macroscopic restoring force were highlighted to play essential roles in generating a giant enhancement in large-signal dielectric permittivity and piezoelectric coefficient^15^. The concept of mechanical dislocation imprinting describes a new paradigm for developing functional materials beyond chemical means (for example, doping^16^ and point defect engineering^17,18^). As the material is doped with its own defects without relying on additional elements, this strategy was also coined as self-doping or sustainable doping^19^. However, dislocation-based anisotropy utilizing 1D-2D defect interactions is still missing, due to the grand challenges of introducing highly oriented dislocations into brittle ceramic oxides^20^ and accurately quantifying their electrical properties, both parallel to dislocations and perpendicular to dislocations.

## Results

### Establishing the basis for 1D-2D defect interaction

1D-2D defect interactions generally can be classified into point-plane (0-dim/2-dim) interaction (a dislocation line intersecting a domain wall) and line-plane (1-dim/2-dim) interaction (a dislocation line embedded in a domain wall), as schematically highlighted in Supplementary Fig. 1. As a result, the pinning force of the 0-dim/2-dim interaction is exerted on an individual intersection, leading to a weak pinning force for domain wall motion. By contrast, the 1-dim/2-dim case entails the complete line intersection with the concentrated pinning force on it, where a maximized domain wall pinning is expected. This domain-wall pinning anisotropy gives rise to large anisotropic functionalities if dislocations are well-aligned. Large anisotropy in properties has been observed in ferroics (for example, domain-engineered ferroelectric crystals^21^), but this behavior stems mainly from the intrinsic crystallographic structure (for example, anisotropic free energy profile for polarization rotation^22^) and not from anisotropic defect interactions. However, a clear picture of the above-predicted domain-wall pinning anisotropy based on geometric line-plane relationships has been lacking. This prevents deploying defect design strategies to engineer multi-dimensional defects of materials.

**Fig. 1:**
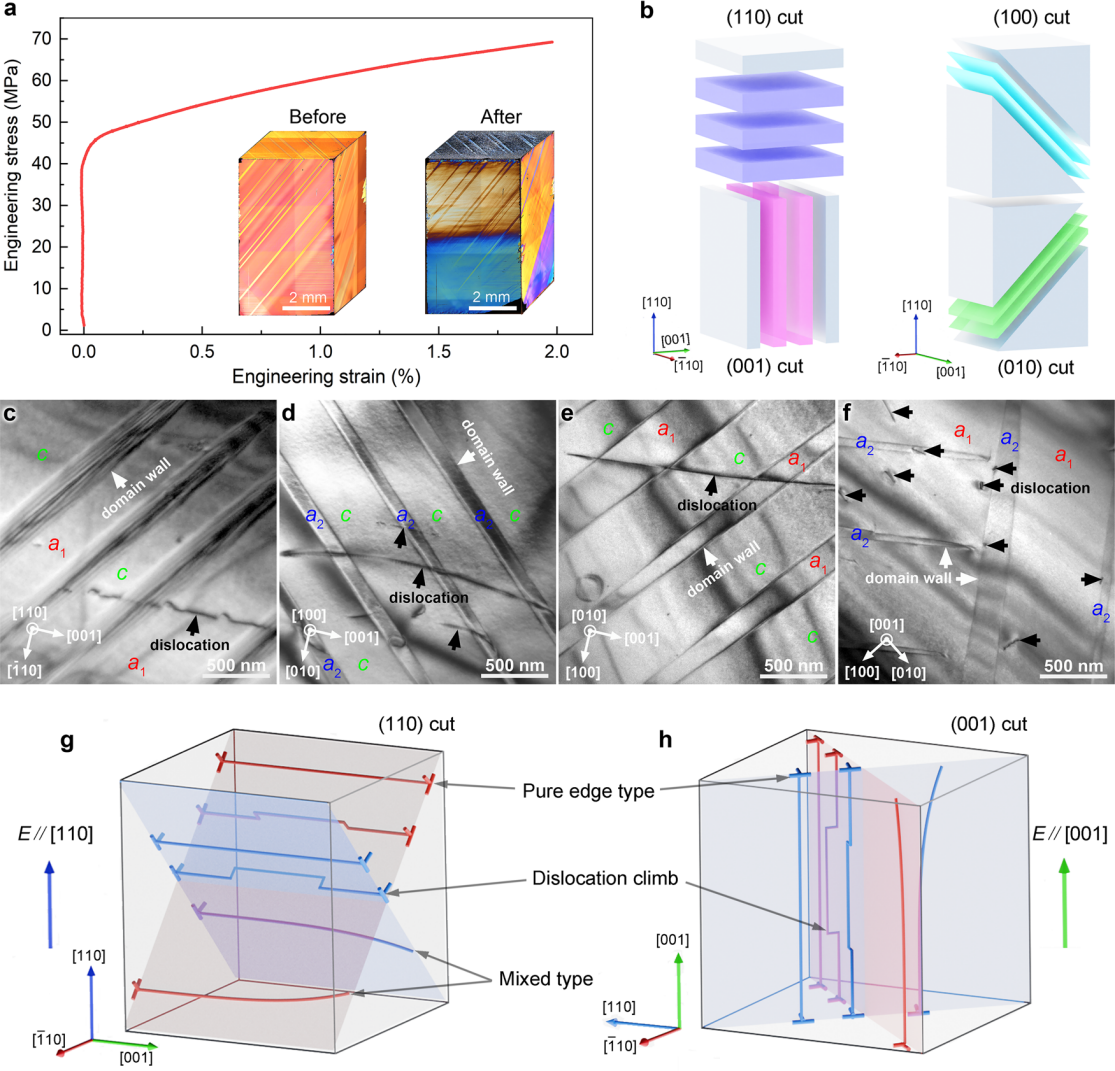
Dislocation structure design using high-temperature bulk deformation and characterization. **a** Stress-strain curve at 1150 °C obtained by uniaxial compressive plastic deformation with a loading-rate of 0.2 N s^–1^. We selected [110]-loading to introduce dislocations as the critical stress for plastic deformation is much smaller than that of [001]-loading, as shown in Supplementary Fig. 2c. The insets reveal the typical domain structure with 90° domain walls before and after deformation. TEM images were taken after preparing samples by slicing the deformed samples into pieces parallel to (110), (100), (010) as well as (001) planes, see extracting slices in **b**. Bright-field TEM images of dislocations that formed in the deformed BaTiO_3_ crystal, when imaging the **c**, (110) plane, (**d**, **e**), (100) and (010) slip planes, and the **f**, (001) plane. Black and white arrows indicate the positions of dislocations and domain walls, respectively. Domains are marked on each TEM image with *a*_1_-domains (polarization vectors parallel to [100]), *a*_2_-domains (polarization vectors parallel to [010]), and *c*-domains (polarization vectors parallel to [001]), see Supplementary Note 1. **g**, **h** Schematic illustration of the {100}<100> slip systems, activated during uniaxial compressive plastic deformation at high temperature, enabling measurements perpendicular and parallel to the dislocation lines in (110)- and (001)-cut samples, respectively. Features of pure edge dislocations, dislocation climb and mixed dislocations were documented.

Here we address the challenges of dislocation-based anisotropic design by uniaxial plastic deformation of the ferroelectric model system BaTiO_3_. Well-chosen single crystal orientations then reveal anisotropic dielectric and electromechanical properties. To introduce directed mesoscopic dislocation structures, BaTiO_3_ crystals were plastically deformed at 1150 °C by loading along the [110] direction, see Methods and Supplementary Fig. 2. A distinct plastic deformation regime in the stress-strain curve (Fig. 1a) marks the introduction of dislocations into BaTiO_3_. As compared to the reference sample, the domain patterns were altered by the mechanical imprint, see Supplementary Fig. 3. Transmission electron microscopy (TEM) images viewed on (110), (100), (010) and (001) sliced samples (Fig. 1b) confirmed the directional control achieved via mechanical dislocation imprint (traces of dislocation lines are identified to run parallel to the [001] direction, as displayed in Fig. 1c–e). TEM images revealed the activation of the {100}<100> slip systems with Burgers vector of ***b*** = [010] (see Supplementary Fig. 4 for details). Note that short dislocation segments along the [100] or [010] direction were observed on the (001) plane (Fig. 1f), which are caused by either dislocation climb or screw partials of mixed dislocations, see discussion in Supplementary Fig. 5. Based on our TEM results, the dislocation spacing was estimated to be 100‒500 nm.

Note that the well-aligned dislocations favor two *a*–*c* domain variants (both *a*_1_–*c* and *a*_2_–*c*) in (110), (100) and (010) cuts, but stabilized the *a*_1_–*a*_2_ variants in the (001)-cut sample (Supplementary Fig. 6). Hence, mechanically imprinted 1D dislocations were inserted into both cuts with an anisotropic 2D domain wall distribution. They yield a platform for disclosing the dislocation-induced anisotropy by quantifying electrical properties perpendicular to dislocations (Fig. 1g) and along the dislocations (Fig. 1h). This suggests that the role of DDW interactions and their effects on dielectric and piezoelectric properties in our material stems from our 1D-2D defect approach. Mathematically, for our case of directional imprint, this signifies an elevation of rank of piezoelectric tensor by combining it with the two-dimensional strain field of the dislocations.

### Anisotropic dielectric and piezoelectric response induced by oriented dislocations

**Fig. 2:**
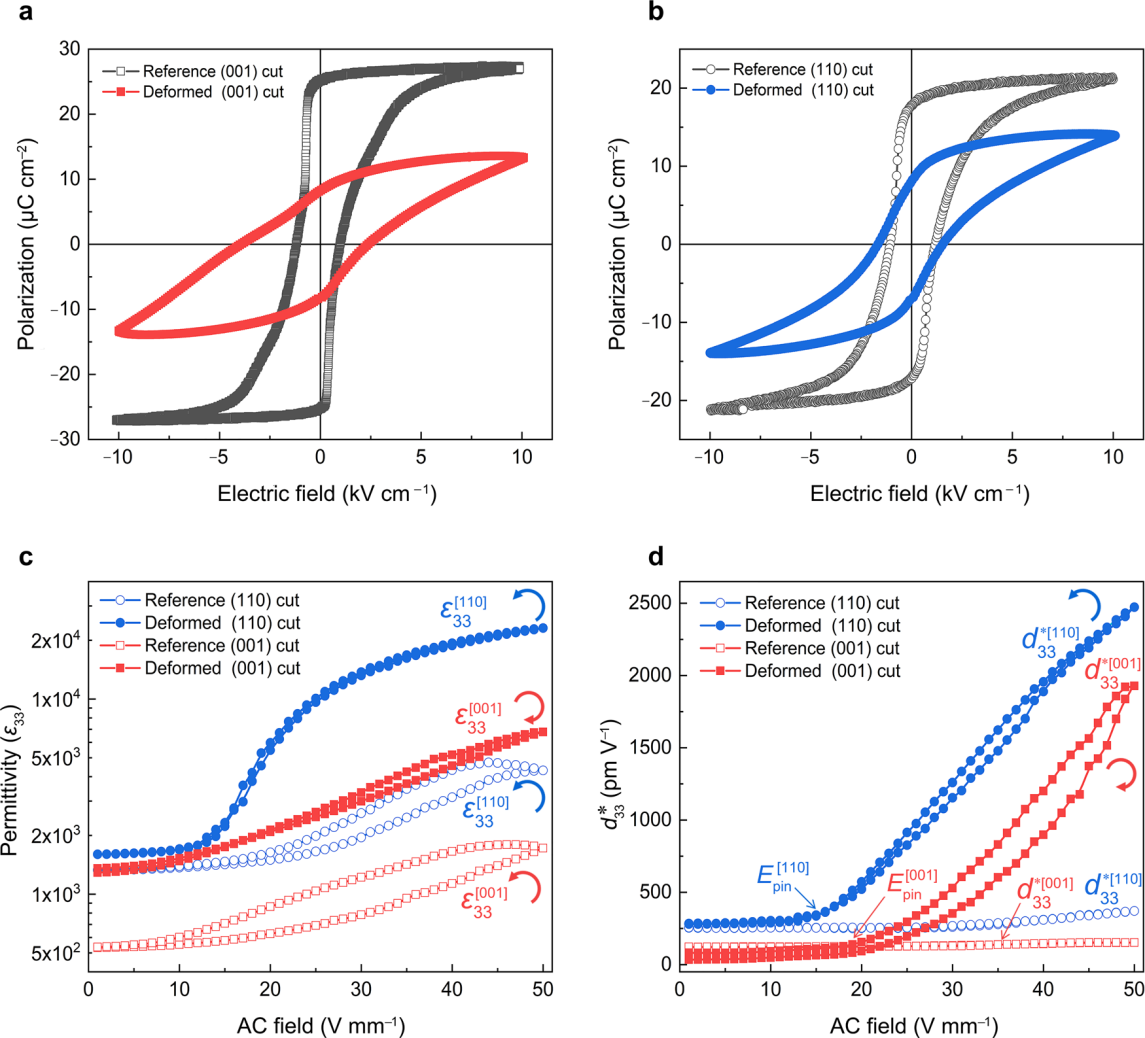
Influence of dislocations on electrical properties. **a**, **b** Polarization hysteresis loops of reference and deformed (001)- and (110)-cut samples quantified at room temperature with a frequency of 1 Hz. **c** Dielectric permittivity, *ε*_33_, and **d** corresponding converse piezoelectric coefficient, *d*_33_*, as a function of the amplitude of AC field for reference and deformed (001)- and (110)-cut samples measured at 1 kHz. The inset arrows indicate that the *ε*_33_ and *d*_33_* exhibit clockwise (red semicircle arrow) and counter-clockwise hysteresis (blue semicircle arrow) during the AC field cycle. The pinning electric field is defined as the point where *d*_33_* starts to increase dramatically.

For didactic considerations, this novel field is best introduced by employing a tetragonal single-crystal ferroelectric with two domain wall variants only. To this end, BaTiO_3_ was selected since both 90° and 180° walls contribute to permittivity and piezoelectricity, and the contribution of 180° walls to piezoelectric coefficient is negligible^23^. The introduced mesoscopic dislocation structure reveals a significant impact on domain switching. Macroscopic measures like a reduced spontaneous polarization and an enhanced coercive field (*E*_c_) (Fig. 2a, b and Supplementary Table 1) can be readily recorded. Domain wall movement in the sub-coercive regime was then quantified employing small-signal excitations (Fig. 2c, d). Both, permittivity (*ε*_33_), but more so converse piezoelectric coefficient (*d*_33_*), reveal an almost constant response at a small alternating current (AC) drive but a dramatic increase beyond a pinning field, see Methods and Supplementary Fig. 7. Remarkably, the (110)-cut deformed sample had higher large-signal sub-coercive values ($${\varepsilon }_{33}^{\left[110\right]}\, \approx \, 23100,$$$${d}_{33}^{*\left[110\right]}\, \approx \, 2470$$ pm V^–1^ at 50 V mm^–1^) than the (001)-cut deformed sample ($${\varepsilon }_{33}^{\left[001\right]}\, \approx \, 6780$$,$${d}_{33}^{*\left[001\right]}\, \approx \, 1930$$ pm V^–1^). Sub-coercive-signal properties of our previous report^15^ in the deformed sample with {101} < 101 >  slip systems (*ε*_33_ ≈ 5800 and *d*_33_*≈ 1890 pm V^–1^) are comparable to the (001)-cut deformed sample, but smaller than that observed in the (110)-cut deformed sample (*d*_33_* is higher than that obtained in the best lead-based perovskite oxides at weak fields^21^, e.g., Pb(Mg_1/3_Nb_2/3_)O_3_-PbTiO_3_ with *d*_33_* ≈ 2000 pm V^–1^). We note that both permittivity and *d*_33_* of the (110)-cut deformed sample, had a counter-clockwise cycle during loading and unloading hysteresis, while for the (001)-cut deformed sample, the hysteresis went clockwise during increasing/decreasing AC field (Fig. 2c, d). As featured in Fig. 2c, d, the anisotropic dielectric and piezoelectric properties of the reference samples are caused by the crystallographic orientation and the anisotropic dielectric tensor of BaTiO_3_ single crystal with *ε*_*a*_ > *ε*_*c*_^24^. The dislocation-tuned anisotropic sub-coercive-signal properties were pronounced in the deformed samples, which is associated with both crystallographic orientation and dislocation-induced effects. Small-signal permittivity of the deformed samples was increased when comparing with the reference samples (Supplementary Fig. 8a, b). The Curie point was slightly increased after deformation, which is caused by the local stress field of the dislocations introduced (see phase-field simulations in Supplementary Fig. 8c). It was reported that interface dislocations can degrade ferroelectric properties^25,26^ or induce large local polarization inhomogeneity of epitaxial films^14^ (for instance, a single **a**[001] dislocation could create a large local polarization inhomogeneity of ~100 μCcm^−2^ in PbZr_0.2_Ti_0.8_O_3_ film^14^.

**Fig. 3:**
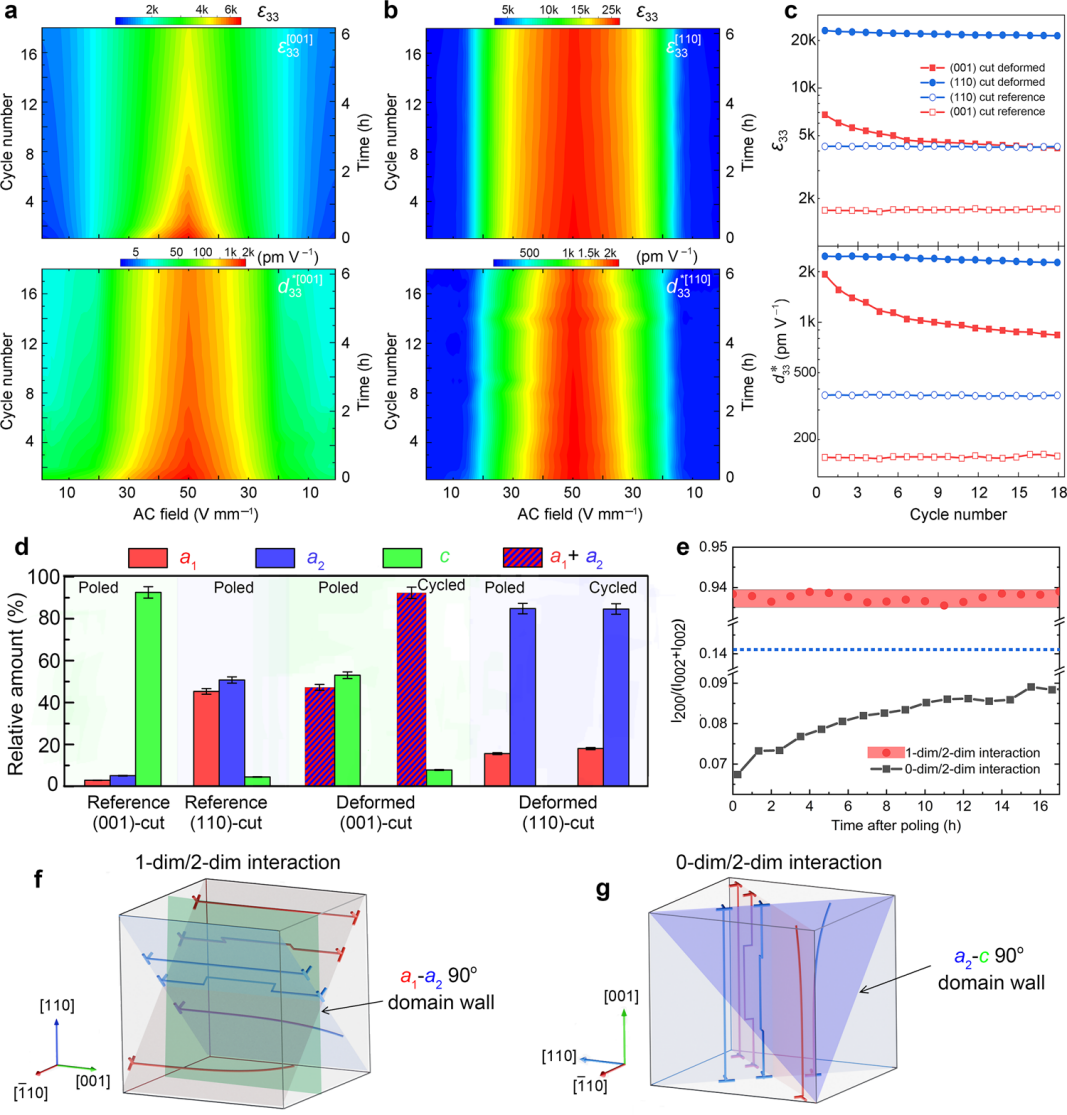
Dislocation-induced stability and anisotropic DDW interactions. Relative dielectric permittivity, *ε*_33_ and piezoresponse, *d*_33_* during cycling measurements at 1 kHz for **a** (001)-cut deformed and **b**, (110)-cut deformed samples. **c** *ε*_33_ and *d*_33_* at 50 V mm^–1^ as a function of cycle number for both (001)- and (110)-cut samples. **d** Comparison of the domain distribution of both (110)- and (001)-cut samples poled at 1 kV mm^–1^, and after cycling, determined from NMR spectra. Green color indicates the *c*-domains with polarization vector pointing toward [001], namely, perpendicular to the original mechanical loading axis. Red and blue colors refer to the *a*_1_-domains (polarization vector pointing toward [100]) and *a*_2_-domains (polarization vector pointing toward [010]). Therefore, for (110)-cut samples, out-of-plane domains refer to *a*_1_ (red) and *a*_2_ (blue), in-plane domains correspond to *c* (green). (001)-cut samples have out-of-plane *c*-domains and in-plane *a*_1_- and *a*_2_-domains. After the cycling experiments for *ε*_33_ and *d*_33_*, NMR data of both (001)- and (110)-cut deformed samples were collected, namely, cycled samples in **d**. Texture analysis of (200) and (002) reflections reveal that the *a*/*c* ratio of the 0-dim/2-dim interaction increased with increasing time after poling, while the ratio of the 1-dim/2-dim interaction remains stable, as compared in **e**. The short dash line indicates the intensity ratio of the (001)-cut deformed sample in unpoled state. The anisotropic DDW interactions for (110)- and (001)-cut deformed samples are schematically featured in **f**, 1-dim/2-dim interaction and **g**, 0-dim/2-dim interaction, respectively.

Counter-clockwise and clockwise sub-coercive hystereses are suggested to indicate a difference in stability during AC field cycling. To this end, the prominent role of DDW interactions and their effects on dielectric and piezoelectric properties were obtained by extending AC field cyclic loading (see Methods). For the (001)-cut deformed sample, both *ε*_33_ and *d*_33_* decreased significantly at the beginning, then stabilized when measuring along dislocations (Fig. 3a). However, *ε*_33_ and *d*_33_* maintained stable values for the (110)-cut deformed sample when applying the field perpendicular to dislocations (and had anti-clockwise hysteresis in Fig. 2c, d), as displayed in Fig. 3b. The anisotropic stability of deformed samples is related to the dislocation-induced effects when contrasting the crystallographic orientation dependence of sub-coercive-signal properties in reference samples, see *ε*_33_ and *d*_33_* as depicted in Fig. 3c and corresponding displacement in Supplementary Fig. 9. To understand the mechanism underlying the anisotropic stability, we characterized the domain evolution of deformed samples in both cuts during the cycling process. As featured in Supplementary Fig. 10, we found that the *a*_2_/*c* domain ratio of the (001)-cut deformed sample increased, leading to an enhanced permittivity at low fields due to the anisotropic dielectric tensor (Supplementary Fig. 11). Both *a*_2_/*a*_1_ domain ratio and permittivity of the (110)-cut deformed sample were stable.

We utilized non-destructive nuclear magnetic resonance (NMR, see Methods and Supplementary Fig. 12) to quantify the relative amount of domain variants of the entire sample in different conditions. NMR results demonstrated that the designed dislocations stabilized *a*_1_–*a*_2_ domain variants of unpoled (001)-cut deformed sample and two *a*–*c* variants (*a*_1_–*c* and dominant *a*_2_–*c*) of unpoled (110)-cut deformed sample (Supplementary Fig. 13), in agreement with optical observations. Interestingly, direct current (DC)-field poling yielded different domain variants, that is, *a*–*c* variants in poled (001)-cut deformed sample and *a*_1_–*a*_2_ variants in poled (110)-cut deformed sample (Fig. 3d). We consider that the interplay of oriented dislocations and anisotropic 2D domain wall distribution can be more significant when experiencing a sub-coercive AC electric field cycling. We found that *a*_1_–*a*_2_ variants kept a rather stable domain ratio when cycling perpendicular to the dislocations, see the (110)-cut cycled sample in Fig. 3d. NMR data indicated that the *a*/*c* domain ratio of the (001)-cut deformed sample, however, increased from ~47/53 to ~92/8 (see Fig. 3d). Furthermore, NMR data collected with a shorter waiting time also supported the similar trend independently (Supplementary Fig. 14). These anisotropic interactions and changes of domain ratio were corroborated using X-ray diffraction (see texture analysis in Fig. 3e and Supplementary Fig. 15).

On the basis of above-mentioned results, we were able to reconstruct a full picture of 1D-2D defect interactions in BaTiO_3_. As featured in Fig. 3f, *a*_1_–*a*_2_ variants ensured 90° domain walls parallel to dislocation lines, suggesting the 1-dim/2-dim interaction in the (110)-cut sample. By contrast, dislocation lines cut across *a*–*c* 90° domain walls with individual intersections, and generated the 0-dim/2-dim interaction in the (001)-cut sample (Fig. 3g). The domain-wall pinning and field-dependent large dielectric and electromechanical response can only be observed when there is a driving force for the motion of domain walls (see Supplementary Fig. 16). The anisotropy that we describe here has three key signatures. First, dislocation-based scattering anisotropy cannot be easily manipulated^4,8^, while here we address 1D-2D defect-induced dielectric and piezoelectric anisotropy, which can be controlled via geometric line-plane relationships. Second, our 1D-2D defect strategy provides a general behavior that is valid in a wide range of other systems and may be implanted independently of crystal geometry, including for example any ferroelectric system with non-180° and 180° domains to tune their di-/ferro-/piezo-/electric properties, or correlated electron oxide systems (for example, multiferroics^26^, superconductors^27^) to harvest versatile functionality. Third, wider perspectives of the 1D-2D defect engineering encompass concepts, such as anisotropic domain-wall engineering^28^ and anisotropy control in dislocation arrangement of ferroics^29^.

### Origin of dislocation-tuned anisotropic functionality

To mechanistically rationalize and quantify the role of anisotropic DDW interactions in dielectric permittivity and piezoelectric response, we calculated the local pinning phenomenon of both 1-dim/2-dim and 0-dim/2-dim interactions based on the phase-field simulations and the generalized theory of configurational forces (see Methods). The mechanical far-field loading activated the {100}<100> slip systems with two perpendicular slip directions, as confirmed by TEM (Fig. 1c and Supplementary Fig. 5). We included here two parallel dislocation lines and perpendicular Burgers vectors as a simple dislocation network to disclose the origin of dislocation-induced functionality anisotropy.

**Fig. 4:**
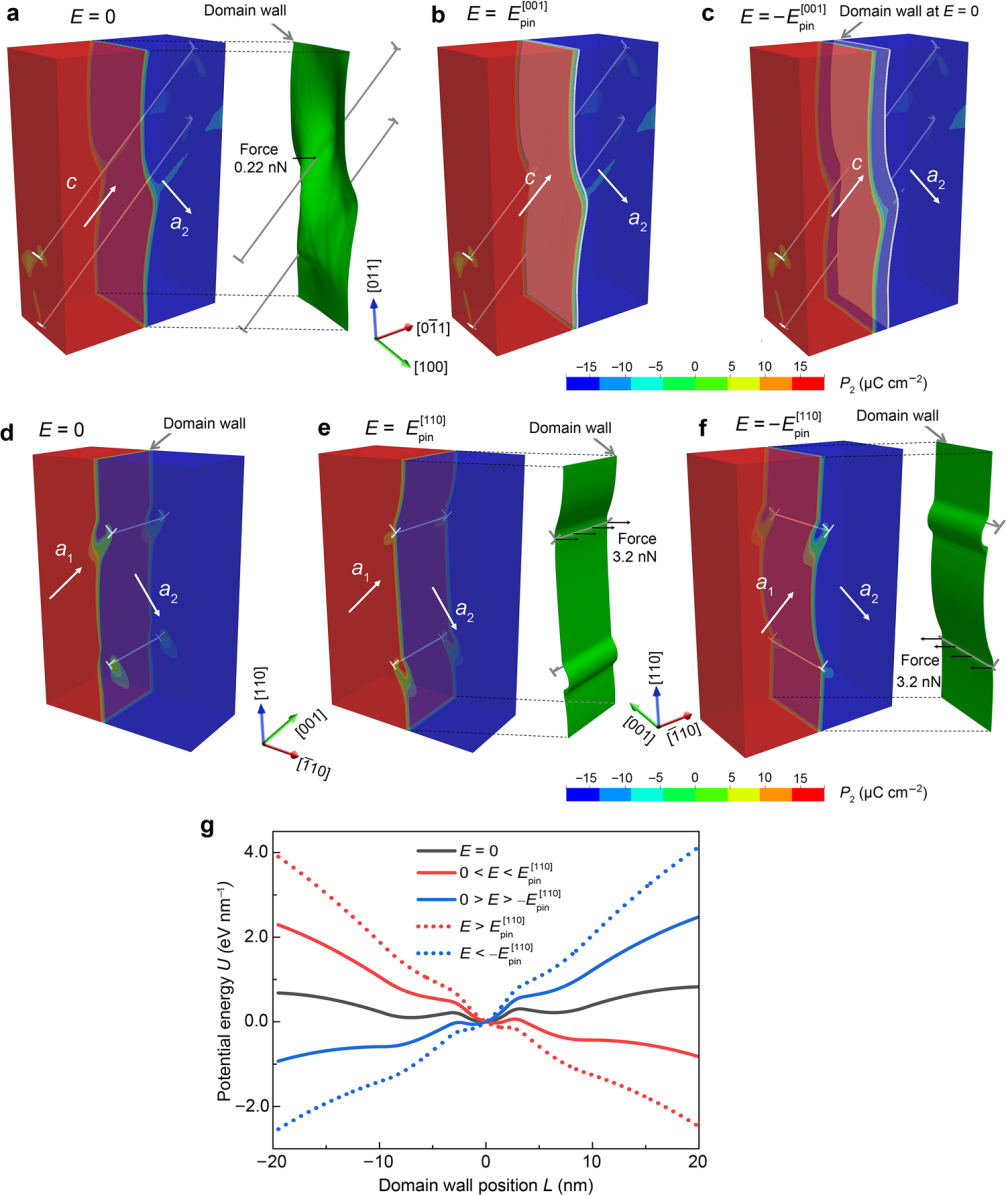
Understanding of anisotropic DDW interactions. Phase-field simulations of the 0-dim/2-dim interaction in (001)-cut deformed sample under different amplitude of AC fields along the [001] direction (that is, parallel to the dislocation line vector), **a** *E* = 0, **b** $E={E}_{{pin}}^{\left[001\right]}$, and **c** $E={-E}_{{pin}}^{\left[001\right]}$. In this case, the local pinning force exerted on the intersection points is much smaller. As a result, the domain wall can be easily moved to the right side of the initial position under $E={E}_{{pin}}^{\left[001\right]}$ in **b**, or the left side of the initial position at $E={-E}_{{pin}}^{\left[001\right]}$ in **c**. Therefore, the 90° domain wall moves towards the $\left[00\bar{1}\right]$ direction when the applied electric field is positive and small ($E\, < \,{E}_{{pin}}^{\left[001\right]}$). The positive electric field prevents the movement of the *a*_2_–*c* domain wall towards the $\left[00\bar{1}\right]$direction. The negative electric field enhances the movement of the 90° domain wall towards the $\left[00\bar{1}\right]$ direction (**c**). For the (110)-cut deformed sample, the 1-dim/2-dim interaction comes into play, as demonstrated in **d** under *E* = 0. In this case, the applied AC field is along the [110] direction (that is, parallel to the domain wall). The 1-dim/2-dim interaction provides a higher local pinning force of 3.2 N exerted on the intersection lines **e** under $E={E}_{{pin}}^{\left[110\right]}$, and **f** under $E={-E}_{{pin}}^{\left[110\right]}$. **g** Corresponding potential energy (*U*) as a function of domain wall position (*L*) for 1-dim/2-dim interaction. The dislocation spacing was set as 200 nm for potential energy calculations.

Figure 4a depicts the 0-dim/2-dim interaction between the *a*_2_–*c* 90° domain wall and the dislocation lines at initial time step. The intersection provides a pinning force of 0.22 nN (corresponding stress is 0.029 MPa) on the domain wall towards the $\left[00\bar{1}\right]$ direction, which is enhanced with applied AC field. Hence, it reduces the amount of *c*-domains and increases the amount of *a*_2_-domains (see Fig. 4b, c and Supplementary Movie 1). In this case, the domain wall has no equilibrium position. Figure 4d features the domain structure of 1-dim/2-dim interaction before poling, where a strong DDW interaction is still not activated. The *a*_1_–*a*_2_ 90° domain wall is located initially on metastable equilibrium positions far away from the dislocations. After poling, the domain wall moves to a stable equilibrium position between the two dislocations with perpendicular Burgers vectors. During AC field cycling, the 90° domain wall remains pinned by one dislocation line, but it depins from the other one easily (see Fig. 4e, f and Supplementary Movie 2). Consequently, the dislocation line provides a strong antisymmetric pinning force of 3.2 nN (corresponding stress is 0.43 MPa) on the motion of 90° domain wall when the wall gets trapped by a pair of dislocations, indicating a stable domain structure during cycling. The anisotropy of the local pinning force is responsible for an anisotropic domain-wall stability, which is consistent with our NMR data (Fig. 3d) and optical observations (Supplementary Fig. 10).

Calculations of the energy profiles indicate that the *a*_1_–*a*_2_ 90° domain wall has three equilibrium positions for the case of *E* = 0 (Fig. 4g). When the amplitude of the AC field is below the pinning field (Fig. 4f), the movement of the domain wall is reversible around the stable equilibrium position in the deep energy well and irreversible around the metastable equilibrium positions in the shallow energy well (Fig. 4g and the dependence of dislocation spacing in calculations in Supplementary Fig. 17). The pinned domain wall depins at an AC field higher than *E*_pin_ to overcome the potential barrier, thereby developing giant irreversible domain wall displacements (can be tens of nanometers). The combined reversible and irreversible movement of the domain wall leads to field-dependent piezoelectric and dielectric properties of the material^30^. The variation of the driving force (Supplementary Fig. 18) and corresponding deep energy well of the 1-dim/2-dim interaction stabilizes *a*_1_–*a*_2_ domain variants and reversible domain wall motion during AC cycling. This suggests that the 1-dim/2-dim interaction generates a giant and stable dielectric and electromechanical response (Fig. 3b). Note that complete reversibility is ensured by the macroscopic restoring force (Supplementary Fig. 19 and Supplementary Note 2) as described by Höfling et al.^15^.

In contrast, for the 0-dim/2-dim interaction case, the domain wall rather moves towards the region of *c* domain under AC field during cycling (the domain wall moves approximately 10 nm from its initial position after one cycle in Fig. 4c). Any increase or decrease of the applied electric field from the neutral equilibrium state triggers the displacement or bending of the domain wall and reduced the amount of *c*-domains. Consequently, there is an increased ratio of *a*_1_–*a*_2_ domains. However, the applied electric field in the [001] direction does not provide driving forces for the motion of *a*_1_–*a*_2_ 90° domain wall (see analytical solution in Eq. (9) and experimental evidence in Supplementary Fig. 16), therefore, it leads to the observed degradation of the field-dependent dielectric and electromechanical response (Supplementary Note 2). By considering two dislocation lines and a 90° domain wall for the (110)-cut deformed sample (see Supplementary Fig. 20), we found that the relative permittivity can reach a value of 10,000 and is dependent on the dislocation configuration and dislocation density. The experimentally observed dielectric and electromechanical response, and pinning field can therefore not be simulated without further knowledge of the exact three-dimensional DDW network interaction. We cannot rule out that the contributions from 180° domain walls to dielectric permittivity come into play, but the contributions are not dominant because our simulations reveal similar 1-dim/2-dim *vs* 0-dim/2-dim interactions for all possible DDW configurations including both 90° and 180° domain walls (Supplementary Fig. 21 and Table 2).

## Discussion

Dislocation networks were mechanically imprinted into BaTiO_3_ using high-temperature plastic deformation, overcoming the long-standing challenge of the introduction of controllable dislocations in bulk ferroelectrics. We developed a general methodology for the targeted use of oriented dislocations for engineering dielectric and electromechanical properties via 0-dim/2-dim and 1-dim/2-dim interactions. Such a defect approach hinges on the concept of the line-plane relationship in mathematics, and serves as an indication of a common mechanism of the DDW interactions. We anticipate that this defect strategy holds for optimizing material functionality covering a range of anisotropies (crystallographic anisotropy, dislocation orientation, domain-wall distribution, DDW configuration), deformation mechanisms and nanomechanics.

Our 1D-2D defect approach is fundamentally different from emerging dislocation engineering^4–9^ that depends on anisotropy of electric or thermal transport. The established constitutive law in this work is not strictly limited to 1D-2D defect systems and may open a door for engineering multidimensional defects with broader perspectives. For example, the 0-dim/2-dim interaction has individual intersection points reminiscent of point defect–domain wall interactions that are capable of tailoring local domain-wall conductivity in ferroelectrics^31^. Dislocation-based domain-wall engineering offers a promising route to the systematic development of ferroelectric materials beyond widely used chemical means including chemical doping-associated polarization rotation (e.g., morphotropic phase boundary^22^) and local structural heterogeneity^16^. In addition, other electronic properties could be anisotropically influenced by dislocations as well. For example, enhanced superconductivity^4^, and ferromagnetic dislocations^30^ have been discovered in antiferromagnetic NiO.

Previous numerical simulations^32–34^ have demonstrated the effects of [110]-type dislocations on the equilibrium position and shape of both ferroelastic 90° and ferroelectric 180° domain walls in ferroelectric single crystals. The pinning force computed for [110]-type dislocations interacting with 180° domain walls is larger than that for dislocations interacting with 90° domain walls. In this work, we reveal that the strength of the pinning force for [100]-type dislocations interacting with both 90° and 180° domain walls strongly depends on the Burgers vector and DDW configuration (see summary in Supplementary Table 2). For example, a weak pinning for the motion of ferroelectric 180° domain walls and a strong pinning for ferroelastic 90° domain walls can be accessible with a precise choice of Burgers vector and DDW configuration. The weak pinning of the 180° domain wall has been experimentally observed in a tetragonal PbZr_0.2_Ti_0.8_O_3_ film with misfit dislocations (Burgers vector [100])^12^, while the strong pinning of the 90° domain wall by a pair of misfit dislocations (Burgers vector $a/2\left[10\bar{1}\right]$) has also been seen in the PbZr_0.2_Ti_0.8_O_3_ film^35^. Therefore, our general line-plane relationship between dislocations and domain walls and pinning force calculations provide a direction to understand and design the complex DDW interactions in thin-film and bulk ferroelectrics. However, the experimentally observed dielectric permittivity and electromechanical response cannot be simulated without knowledge of the exact three-dimensional defect distribution of dislocations and domain walls. This calls for advanced transmission electron microscopy^14,36,37^ to image the polarization and charge distribution around the dislocation cores or dark-field X-ray microscopy^38^ to track the complex DDW interactions. Our simulations revealed that the impact of positive and negative charges on the domain-wall pinning from a single dislocation core is relatively small (Supplementary Fig. 22). In addition, the “charge” effect due to flexoelectricity is indeed possible for edge dislocations in BaTiO_3_^39^. However, the flexoelectric part contributed from the first order term in the Taylor expansion of the strain components, in general, should be much smaller than the strain components from dislocations. A recent experimental and numerical study revealed that the enhancement of polarization around the dislocation core is dominated by the strain rather than the strain gradient^14^. Thus, we believe that the influence of flexoelectricity on the pinning strength of the dislocation is of secondary nature.

The computed pinning strengths for the interactions between [110]-type dislocation arrays and 90° and 180° domain walls are enhanced with increasing dislocation density^33^. Our phase-field calculations suggest that the pinning field for [100]-type dislocations interacting with the 90° domain wall increases with increasing dislocation density (see Supplementary Fig. 20c). The dislocation density itself is dependent on the experimental loading parameters, such as loading rate, stress, strain, and temperature^1^. In this work, the introduced dislocation density is estimated as ~2 × 10^12^ m^–2^, by quantifying the observed TEM images. The pinning field for the (110)-cut deformed samples cut from a single deformation and obtained from different deformations with the same loading parameters is ~16 V mm^–1^ (see Supplementary Fig. 23), indicating that the dislocation density is in the order of ~2 × 10^12^ m^–2^. To estimate the impact of dislocation density on properties, we additionally deformed a notched sample with dense and ordered [100]-type dislocations (Supplementary Fig. 24a, b). With a dramatic increase in dislocation density (roughly one order of magnitude), the dislocations could severely pin the motion of domain walls, leading to reduced dielectric and piezoelectric properties (Supplementary Fig. 24c, d).

## Methods

### High-temperature deformation and sample preparation

Top seeded solution grown (TSSG) [110]-oriented high-quality BaTiO_3_ single crystals (coordinate system: *X*:$\left[\bar{1}10\right]$; *Y*: [001]; *Z*:[110]) with a geometry of 4 × 4 × 8 mm³ (Electro-Optics Technology GmbH, Idar-Oberstein, Germany) were deformed at 1150 °C in uniaxial compression to activate the {100} <100> high temperature slip system with a Schmid factor of 0.5^40,41^. In this case, the maximized Schmid factor as quantification for the propensity of dislocation slip on the (100) planes leads to generation of dislocations along [001] (see Fig. 1c–f). During heating with 1 °C min^–1^ and thermal equilibration at 1150 °C for 30 min, a pre-load of 1.25 MPa was applied. Compression was conducted at 1150 °C with a loading rate of 0.2 N s^–1^ (0.0125 MPa s^–1^) using a load-frame (Z010, Zwick/Roell, Ulm, Germany) equipped with a linear variable differential transformer (LVDT) for precise displacement measurement. After reaching 2% deformation, the sample was unloaded with a loading rate of 0.5 N s^–1^ (0.031 MPa s^–1^) to avoid barreling of the sample. Afterwards, the sample was cooled down to room temperature with a ramp of 1 °C min^–1^ under a uniaxial compressive stress of 1.25 MPa. Details of the deformation experiments can be found in Supplementary Fig. 2. To study the influence of dislocation density on properties, a notched BaTiO_3_ crystal (Supplementary Fig. 22a) was deformed using the same loading parameters. In this case, the [100]-type dislocations with a high density align preferably in glide planes (see the slip traces in Supplementary Fig. 22a, b) with an angle of 45° to the notch.

To assess anisotropic behavior, deformed crystals were sliced into smaller pieces (Fig. 1b) using a Diamond Wire Saw (Model 4240, Well Diamond Wire Saws, Inc., Le Locle, Switzerland). The orientation of the as-prepared samples was confirmed using Laue back-reflection (1001 Model, Huber, Rimsting, Germany). The surfaces of the (001)- and (110)-cut samples were then finely polished to a thickness of 0.5–1.0 mm. Gold electrodes were sputtered on the two large top and bottom surfaces of the investigated (001)- and (110)-cut samples, and then annealed at 200 °C for 2 h (heating/cooling rate: 1 °C min^–1^) prior to electrical characterization. DC-poling of reference and deformed samples was performed under 1 kV mm^–1^ for 10 min at room temperature.

Bright-field TEM and scanning transmission electron microscopy (STEM) images were taken with a JEM-2100F TEM (JEOL, Tokyo, Japan). The deformed crystal was cut parallel to (110), (100), (010), as well as (001) planes into small pieces with a 300-μm thickness and polished using a MultiPrep polishing system (Allied High Tech Products Inc., Compton, CA, USA) down to 20 μm. To remove any influence from polishing on the dislocations imprinted by high-temperature deformation, the as-polished thin slices were annealed at 200 °C for 30 min with a slow heating/cooling rate of 1 °C min^–1^, to completely release the strain from polishing procedure^40^. The annealed TEM slices were mounted on supporting molybdenum grids of 100 mesh (Plano, Wetzlar, Germany) and thinned by Ar ions using a dual ion milling system (Gatan, Pleasanton, CA, USA) into electron transparency.

### Electrical measurements

Small-signal dielectric permittivity as a function of temperature was quantified with an applied AC field of 1 V (root-mean-square) using a HP 4192 A impedance analyzer (Hewlett Packard, Palo Alto, California, USA) equipped with a furnace (Nabertherm Inc., Lilienthal, Germany) with a heating ramp of 1 °C min^–1^. Polarization hysteresis (*P*–*E*) loops at room temperature were recorded at 1 Hz using a TF 2000E ferroelectric workstation (aixACCT Systems Inc., Aachen, Germany). Sub-coercive AC field dependence of permittivity was obtained using a lock-in amplifier (SR830, Stanford Research System, Sunnyvale, USA) in combination with a high voltage amplifier (PZD700A M/S, Trek Inc., Lockport, USA) generated peak AC voltage and current of ±700 V and ±200 mA, respectively. To simultaneously quantify the converse piezoelectric coefficient, *d*_33_*, we combined the driving-voltage setup with a laser vibrometer (VDD-E-600 PC-Based Digital Vibrometer Front-End and OFV-505 Sensor Head, Polytec GmbH, Waldbronn, Germany). AC voltage-induced strain and displacement signals through the converse piezoelectric effect were determined by the Doppler effect with a high precision of ±1 pm^42^. However, the real resolution of the laser vibrometer is limited by background noise (10–20 pm). The cycling experiment in Fig. 3a, b was performed at 1 kHz by loading the amplitude of AC field from 1→50→1 V mm^–1^ up to 18 cycles (about 6 h for the whole measurement). The developed displacement as a function of time under different AC voltages was recorded using a self-developed Macro code in the VibSoft-VDD software.

### Dislocation and domain structure characterization

Optical images (see insets in Fig. 1a and Supplementary Fig. 3) of the reference and deformed BaTiO_3_ single crystals were taken using a LEXT laser scanning microscope (OLS4100, Olympus, Shinjuku, Japan). Both differential interference contrast (DIC) mode and polarized light mode were used to image domain patterns. For in situ domain observation, the top surface of the sample was additionally polished to optical grade. A transparent gold electrode was sputtered onto the top surface. Domain structures in Supplementary Fig. 10 were documented using an Axio Imager2 microscope (Carl Zeiss, Oberkochen, Germany) equipped with a Linkam stage (HFS600E-PB4, Linkam Scientific Instruments, Tadworth, UK). We used the reflection mode to image domains. Additionally, amplifiers were connected to the Linkam stage for applying AC voltage and recording the field dependence of permittivity.

Bright-field TEM/STEM images of imprinted dislocations were taken together with corresponding selected area electron diffraction (SAED) patterns from the adjacent area. Evaluation of the diffraction patterns revealed that the trace of dislocation lines is mainly parallel to the [001] direction. Two beam conditions were adopted to determine the direction of the Burgers vector of dislocations, demonstrating that ***b*** is perpendicular to ***g*** when ***g*** is parallel to the dislocation line (in Supplementary Fig. 5h, i, ***g*** = $00\bar{2}$ was used). Therefore, ***b*** is determined as [100] or [010] for the {100} <100> high temperature slip systems.

^137^Ba NMR spectra on single crystals (unpoled, poled and cycled) were collected with a Bruker Avance III HD spectrometer (Bruker, Billerica, Massachusetts, USA) equipped with a wide bore 14.1 T Oxford magnet. A single-axis goniometer NMR probe (NMR Service, Erfurt, Germany) with nominal resolution of 0.1° was tuned to 66.71 MHz. An angle of 0° represents the normal vector of the sample holder as parallel to the magnetic field B_0_. An angle of 20° was set to measure samples exposing a (001) face or a (110) face, respectively. A Hahn-echo sequence with τ = 30 µs and a recycle delay time of 1 s for an acquisition time of 0.05 s was applied. The duration of the 90° pulses was set to 3.5 μs. The number of scans was set to 10240, for a used sample volume of about 4 × 4 × 1 mm^3^. The pre-scan delay was set to 10 μs. The chemical shift scale was referenced with respect to a 1 M solution of BaCl_2_ (0 ppm).

X-ray diffraction (XRD) experiments were carried out using a Bruker D8 diffractometer (Bruker Corporation, Karlsruhe, Germany) in Bragg-Brentano geometry using Cu-K_α1,2_ radiation. We used (001)-cut deformed samples (about 4 × 4 × 1 mm^3^) for XRD experiments on two large (001) faces. XRD patterns as a function of time after poling were recorded to determine the domain ratio (or texture analysis) based on the intensity around (200) and (002) peaks. When the sample was poled along [001] and [110], the poling induced *a*–*c* and *a*_1_–*a*_2_ 90° domain wall variants (Supplementary Fig. 15a), respectively. It means that in-plane poling along [110] produced the 1-dim/2-dim interaction with dislocations parallel to the domain wall, which is the same configuration with the (110)-cut deformed sample.

### Phase-field simulations and driving force calculations

Based on our recently established simulation framework^34,43^ combining a ferroelectric phase-field model, non-singular solution of dislocations and the extended configurational force theory, we investigate the anisotropic DDW interactions here. For the ferroelectric phase-field model, the free energy of the BaTiO_3_ single crystal is given in the following

1 $$H=	\int\limits_{V}h{{{{{\rm{d}}}}}}V=\int\limits_{V}\left\{{\alpha }_{{ij}}{P}_{i}{P}_{j}+{\alpha }_{{ijkl}}{P}_{i}{P}_{j}{P}_{k}{P}_{l}+{\alpha }_{{ijklmn}}{P}_{i}{P}_{j}{P}_{k}{P}_{l}{P}_{m}{P}_{n}\right.\\ 	+{\alpha }_{{ijklmnpq}}{P}_{i}{P}_{j}{P}_{k}{P}_{l}{P}_{m}{P}_{n}{P}_{p}{P}_{q}+\frac{1}{2}{c}_{{ijkl}}\left({\varepsilon }_{{ji}}-{\varepsilon }_{{ji}}^{P}-{\varepsilon }_{{ji}}^{D}\right)\left({\varepsilon }_{{kl}}-{\varepsilon }_{{kl}}^{P}-{\varepsilon }_{{kl}}^{D}\right)-\frac{1}{2}{K}_{{ij}}{E}_{i}{E}_{j}\\ 	\left. -{P}_{i}{E}_{i}+\frac{1}{2}{g}_{{ijkl}}{P}_{i,j}{P}_{k,l}\right\}{{{{{\rm{d}}}}}}V$$

where *α*_*ij*_, *α*_*ijkl*_, *α*_*ijklmn*_ and *α*_*ijklmnpq*_ are the phenomenological Landau-Devonshire coefficients. Only *α*_*ij*_ are linearly dependent on temperature, ${\alpha }_{{ij}}=(T-{T}_{C})$, where *T* is the temperature, *T*_C_ is the Curie temperature. _*Pi*_ is the polarization. *c*_*ijkl*_ is the elastic stiffness tensor, ${\varepsilon }_{{ij}}=({u}_{i,j}-{u}_{j,i})/2$ is the total strain defined as the symmetric part of the displacement gradient *u*_*i,j*_. ${\varepsilon }_{{ij}}^{P}={Q}_{{ijkl}}{P}_{k}{P}_{l}$ is the eigenstrain induced by the polarization, where *Q*_*ijkl*_ are the electrostrictive coefficients. ${\varepsilon }_{{ij}}^{D}=({b}_{i}{n}_{j}+{b}_{j}{n}_{i})W({{{{{\boldsymbol{x}}}}}},\, \varpi )/2$ is the eigenstrain of dislocations, where *b*_*i*_ is the Burgers vector, *n*_*j*_ is the normal vector of the slip plane, and $W\left({{{{{\boldsymbol{x}}}}}},\, \varpi \right)$ is the distribution function of the eigenstrain formulated based on a non-singular continuum dislocation theory with a parameter ϖ representing the dislocation core width^15^.${\varepsilon }_{{ij}}^{D}$ is the eigenstrain of dislocations^15^. ${K}_{{ij}}={\omega }_{0}\kappa {\delta }_{{ij}}$ are the dielectric tensor, where *ω*_0_ is the dielectric permittivity of vacuum and *κ* is the relative dielectric permittivity of the bulk. The electric field is defined as ${E}_{i}=-{\varphi }_{,i}$, where *φ* is the electric potential. *g*_*ijkl*_ are the gradient energy coefficients. The material parameters can be found in refs. 15, 44.

The evolution of domain structures is governed by the following equations of motion:

2 $${\sigma }_{{ij},j}+{f}_{i}=0$$

3 $${D}_{i,i}-q=0$$

4 $${\dot{P}}_{i}=-M\frac{\delta H}{\delta {P}_{i}}$$

where *f*_*i*_ is the body force, *q* is the volume charge density, and *M* is the mobility parameter. The stress (*σ*_*ij*_) and electric displacement (*D*_*i*_) are defined through the constitutive relations

5 $${\sigma }_{{ij}}={c}_{{ijkl}}({\varepsilon }_{{kl}}-{\varepsilon }_{{kl}}^{P}-{\varepsilon }_{{kl}}^{D})$$

6 $${D}_{i}={K}_{{ij}}{E}_{j}+{P}_{i}$$

The driving force on the domain wall *F*_*k*_ is calculated based on the generalized theory of configurational forces^34^

7 $${F}_{k}=\int\limits_{V}{\sum }_{{kj},j}{{{{{\rm{d}}}}}}V$$

8 $${\sum }_{{kj}}=h{\delta }_{{kj}}-{\sigma }_{{ij}}{\beta }_{{ik}}+{D}_{j}{E}_{k}-{g}_{{ijmn}}{P}_{m,n}{P}_{i,k}$$

where *V* is the volume of the sample, Σ_*kj*_ is the Eshelby stress tensor, *δ*_*kj*_ is the Kronecker delta, and ${\beta }_{{ik}}={u}_{i,k}-{b}_{i}{n}_{k}W({{{{{\boldsymbol{x}}}}}},a)$, thereby $W({{{{{\boldsymbol{x}}}}}},\, a)$ is the regularization of the Burgers vector^34^. In the finite element simulation, the nodal configurational force on the *I*-th node is computed in the postprocessing ${G}_{i}=\mathop{\bigcup }\limits_{e=1}^{{n}_{{el}}}{\int }_{{Ve}}{\sum }_{{ij}}{N}_{,j}^{I}{{{{{\rm{d}}}}}}V$, where *n*_*el*_ is the number of elements around a node, *V*_*e*_ is the volume of an element, and ${N}_{,j}^{I}$ is the gradient of the shape function. Then, the driving force is calculated by summing up the negative of *G*_*i*_ around the defects. The configurational force theory mentioned above is used to calculate the dislocation induced driving force on the domain wall. Considering a vertical domain wall to move in the *x*_1_ direction, we assume the external electric field induced driving force is superposed on the dislocation-induced driving force for simplicity

9 $${F}_{1}={F}_{1}^{D}+{F}_{1}^{E}={F}_{1}^{D}+2{P}_{2}{E}_{2}^{0}l$$

where${F}_{1}^{D}$ and ${F}_{1}^{E}$ are the driving forces due to dislocation and external electric field, ${E}_{2}^{0}$ is the external electric field in the *x*_2_ direction and *l* is the height of the sample, respectively. The corresponding potential energy of the driving force is calculated as

10 $$U=-{\int }_{0}^{{L}}{F}_{1}{{{{{\rm{d}}}}}}{x}_{1}$$

where *L* is the distance between the domain wall and dislocation and the domain wall is placed perpendicular to the *x*_1_ axis. For a mixed dislocation, the screw component has Burgers vector ***b*** and sense vector ${{{{{\boldsymbol{\xi }}}}}}$ along the *x*_3_ direction. According to the Peach-Koehler force $F=\left({{{{{\boldsymbol{\sigma }}}}}}\cdot {{{{{\boldsymbol{b}}}}}}\right)\times {{{{{\boldsymbol{\xi }}}}}}$^34,45^, the pinning force on the domain wall due to the screw dislocation is *F* = 0. This theoretical estimation means that the interaction between the screw dislocation and the domain wall should be weak and is thus not considered in our simulations.

The simulation used material parameters at room temperature (25 °C). The sample size for three-dimensional simulations in Fig. 4 is 50 × 100 × 150 nm^3^. The periodic boundary condition was applied for the top and bottom surfaces, as well as for the front and back surfaces. The left and right surfaces are traction-free and the applied surface charge density is consistent with the horizontal component of the spontaneous polarization. Considering the boundary conditions, we used the following coordinate systems: (110)-cut deformed sample with *x*_1_:$\left[\bar{1}10\right]$; *x*_2_: [001]; *x*_3_:[110], and (001)-cut deformed sample with *x*_1_:$\left[0\bar{1}1\right]$; *x*_2_: [100]; *x*_3_:[011]. The AC field with amplitude of *E*_pin_ was applied for (110)-cut deformed sample and (001)-cut deformed sample along the $\left[\bar{1}10\right]$and [001] directions, respectively. In Supplementary Fig. 19, the dislocation induced driving force was computed without the external electric field, where the vertical dislocation spacing of 200 nm (based on TEM images) was used. Two-dimensional simulations under the plane strain assumption was considered. Then, the driving force was used to calculate the energy landscape in Fig. 4g using Eqs. (9) and (10). The phase-field model was numerically implemented using finite element method in the open-source software Multiphysics Object-Oriented Simulation Environment (MOOSE)^46^. Numerical simulations were carried out on the High Performance Lichtenberg Cluster at Technical University of Darmstadt.

### Reporting summary

Further information on research design is available in the Nature Research Reporting Summary linked to this article.

## Supplementary information

Supplementary Information

Description of Additional Supplementary Files

Supplementary Movie 1

Supplementary Movie 2

Lasing Reporting Summary

## Data Availability

The data that support the findings of this study are available from the corresponding author upon reasonable request.

## Code availability

The open-source MOOSE framework can be accessed in GitHub [https://github.com/idaholab/moose]. The codes used for the data acquisition and analysis included in the current study are available from the corresponding author upon reasonable request.
