# Supplementary material for: Anisotropic dislocation-domain wall interactions in ferroelectrics
Source: Nat Commun. 2022 Nov 5;13:6676. doi: 10.1038/s41467-022-34304-7 (PMC9637100; doi:10.1038/s41467-022-34304-7)
Supplement: Supplementary file 1 — Supplementary Information [file 41467_2022_34304_MOESM1_ESM.pdf]

Supplementary Information for

Anisotropic dislocation-domain wall interactions in ferroelectrics

Fangping Zhuo[1], Xiandong Zhou[1], Shuang Gao[1,2], Marion Höfling[3], Felix Dietrich[4], Pedro B. Groszewicz[5], Lovro Fulanović[1], Patrick Breckner[1], Andreas Wohninsland[1], Bai-Xiang Xu[1], Hans-Joachim Kleebe[1], Xiaoli Tan[6], Jurij Koruza[7], Dragan Damjanovic[8], Jürgen Rödel[1*]

†Affiliations:

[1]Department of Materials and Earth Sciences, Technical University of Darmstadt, 64287 Darmstadt, Germany

[2]Key Laboratory of Advanced Technologies of Materials (Ministry of Education), School of Materials Science and Engineering, Southwest Jiaotong University, Chengdu 610031, PR China

[3]Department of Physics, Technical University of Denmark, 2800 Kgs. Lyngby, Denmark

[4]Institute of Physical Chemistry, Technical University of Darmstadt, 64287 Darmstadt, Germany

[5]Department of Radiation Science and Technology, Delft University of Technology, Delft 2629JB, Netherlands

[6]Department of Materials Science and Engineering, Iowa State University, Ames, IA 50011, USA

[7]Institute for Chemistry and Technology of Materials, Graz University of Technology, A-8010 Graz, Austria

[8]Institute of Materials, École Polytechnique Fédérale de Lausanne, 1015 Lausanne, Switzerland

*Corresponding author: roedel@ceramics.tu-darmstadt.de

This file includes:

Supplementary Figures 1-24

Supplementary Tables 1-2

Supplementary Notes 1-2

Supplementary References

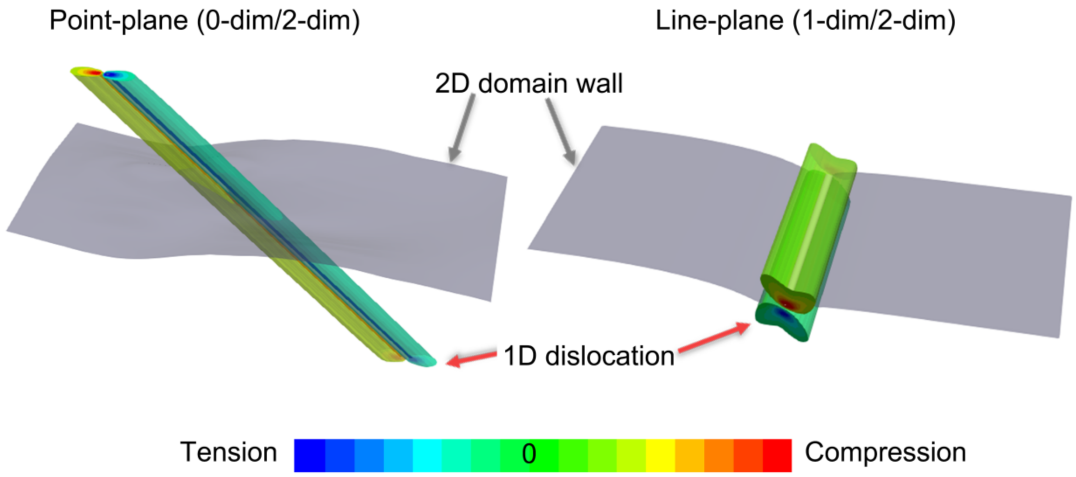

Supplementary Figure 1. 0-dim/2-dim interaction *vs* 1-dim/2-dim interaction between a dislocation line and a domain wall. Schematics demonstrating the elastic stress field around an edge dislocation line and dislocation-domain wall configurations. In the 0-dim/2-dim interaction, the line cuts through the plane at an intersection, while in the 1-dim/2-dim interaction, the line is embedded in the plane.

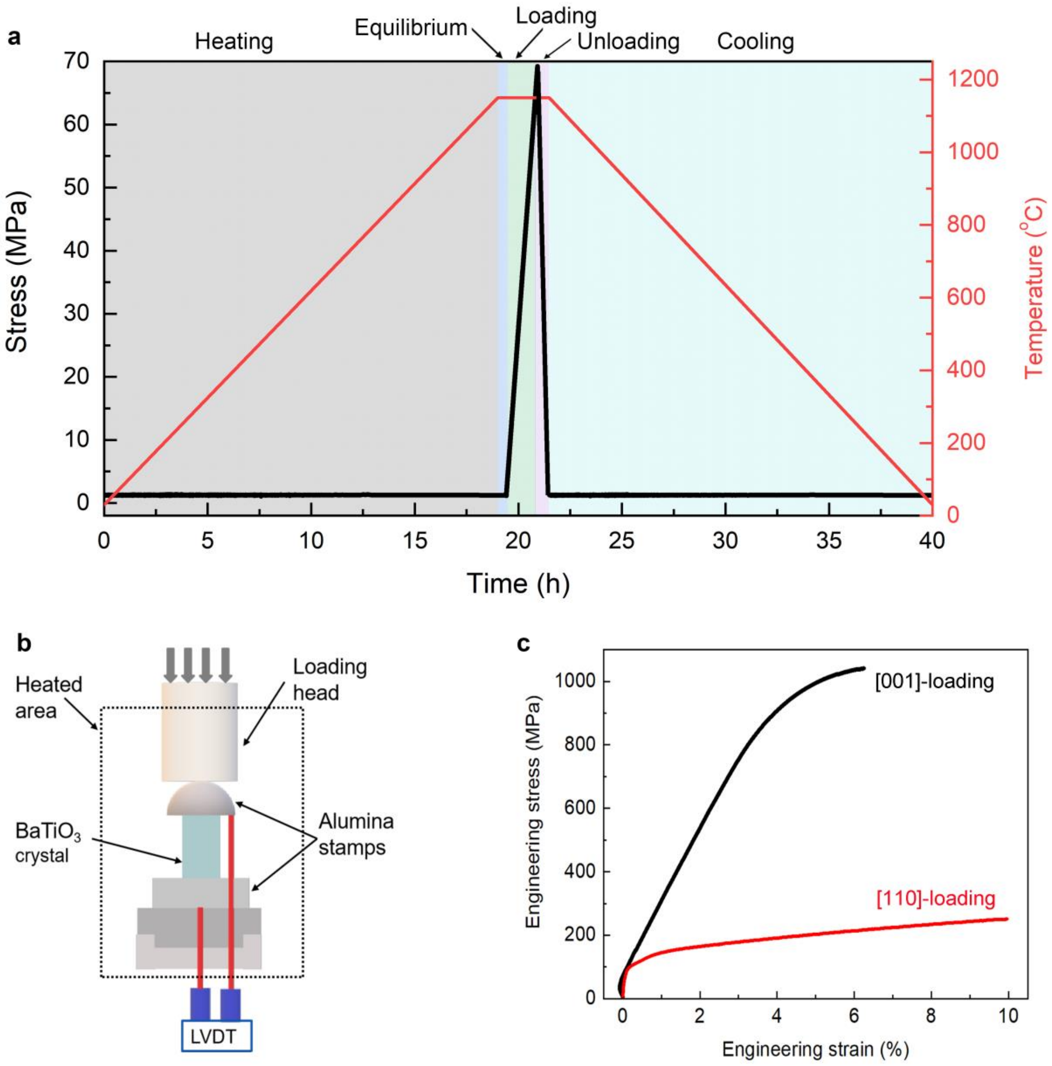

Supplementary Figure 2. High-temperature uniaxial compressive deformation. a, The high-temperature deformation experiment was divided into 5 steps: heating from room temperature to 1150 °C under a constant compressive preload stress of about 1.25 MPa, establishing thermal equilibrium, loading with a loading rate of 0.2 N s^{-1} (0.0125 MPa s^{-1}), unloading with a rate of 0.5 N s^{-1} (0.03 MPa s^{-1}), and cooling down to room temperature under a constant preload stress of about 1.25 MPa. The whole high-temperature deformation experiment took about 40 h. **b**, Schematic depicting the load frame during uniaxial compressive loading. The $BaTiO_3$ single crystal was placed between two alumina stamps. To ensure a homogenous stress distribution during uniaxial compression process, a centering tool was used for the alignment of the $BaTiO_3$ single crystal. **c**, Comparison between [110]-loading and [001]-loading with a rate of 2 N s^{-1} at 1150 °C. Note that the stress-strain curves in **c** were obtained from samples with dimension of $2 \times 2 \times 4$ mm^3.

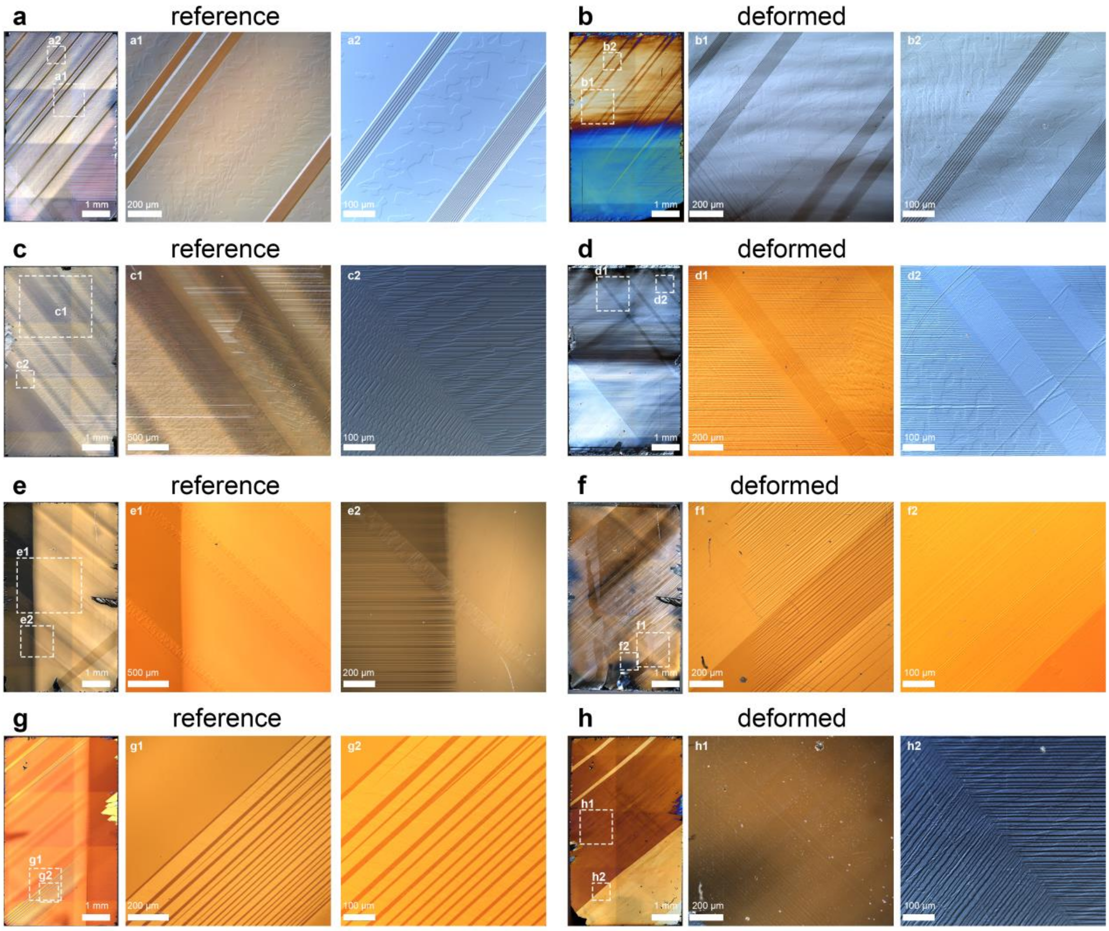

Supplementary Figure 3. Optical images of reference and deformed [110]-oriented tetragonal $BaTiO_3$ single crystals taken at room temperature. (**a**, **c**, **e**, **g**) Domain patterns on side surfaces of the undeformed reference sample. (**b**, **d**, **f**, **h**) Domain patterns on side surfaces of the deformed sample. Under optical microscopy, typical domain structure with 90° domain walls is visible in both reference and deformed samples.

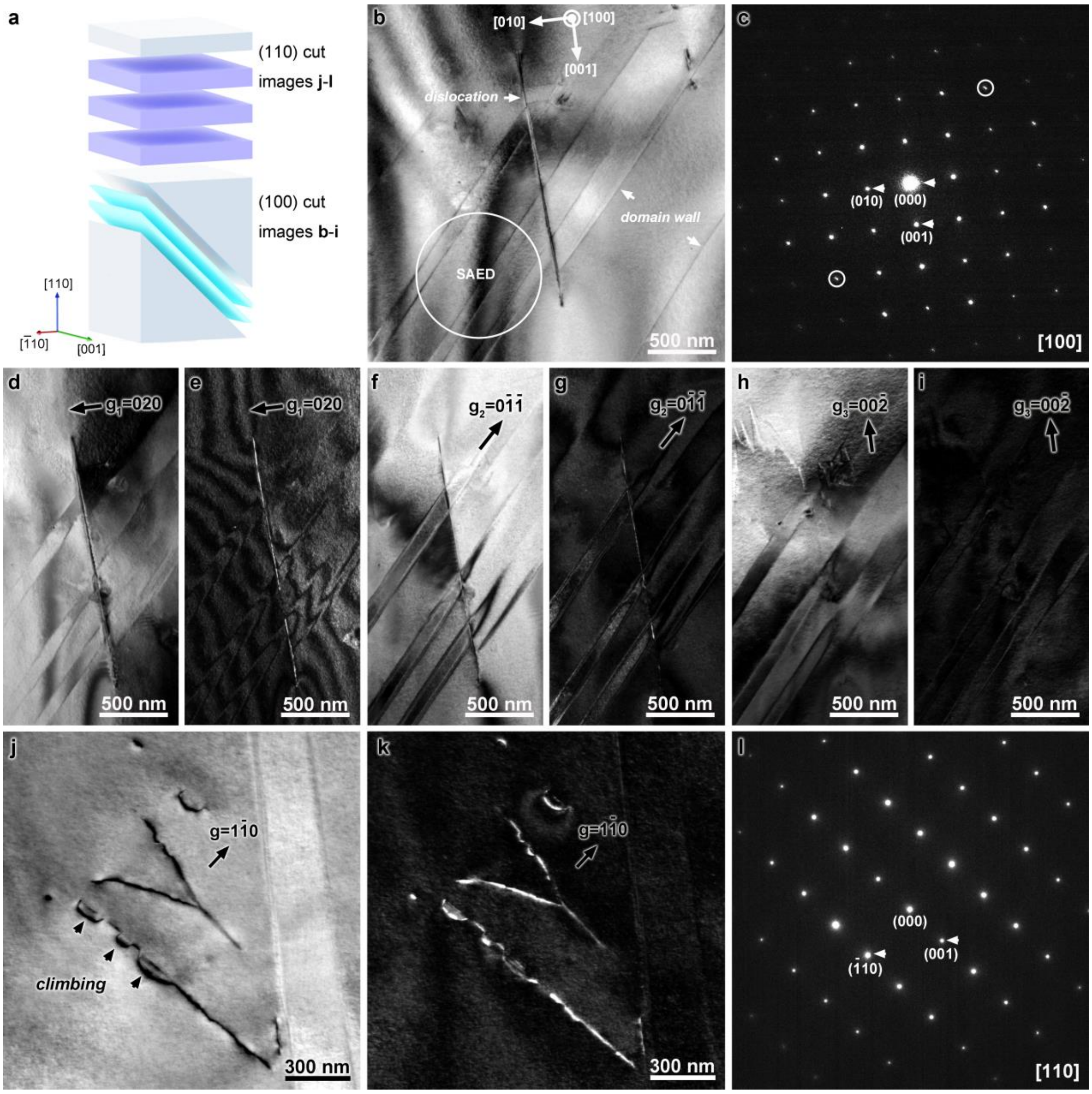

Supplementary Figure 4. Detailed visualization of dislocation structures. a, Schematic illustration of the extracted slices for TEM experiments viewed on (110) plane and (100) slip plane. **b**, Bright-field TEM image of edge dislocation and edge-on domain walls viewed on (100) plane and **c**, SAED pattern obtained in the area marked with white circle in **b**. Splitting of high order reflections of (022) and (0$\bar{2}\bar{2}$) as circled in **c** demonstrates the presence of edge-on 90° domain walls in **b**. Determination of the Burgers vector of the edge dislocation viewed in **b** was by bright-field (**d**, **f**, **h**) and ***g***/3***g*** weak beam dark-field (**e**, **g**, **i**) images with three different ***g*** vectors. The straight dislocation line has a Burgers vector of ***b*** = [010]. Images in **j**, **k**, and **l** depict the dislocation climbing with a typical half-scallop morphology, in which **j**, bright-field and **k**, ***g***/3***g*** weak beam dark-field were viewed on (110) plane and taken with ***g*** = $1\bar{1}0$, corresponding SAED pattern at [110] axis is given in **l**.

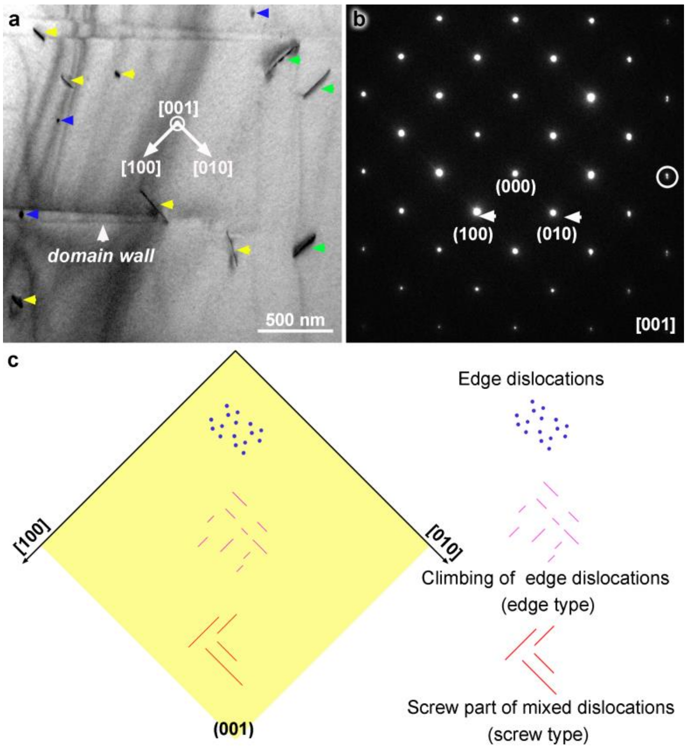

Supplementary Figure 5. Dislocations viewed on (001) plane. A representative bright-field TEM image of the deformed $BaTiO_3$ sample is provided in **a**, with the positions of dislocations marked by arrows. Blue, green and yellow arrows indicate pure edge dislocations and short dislocation segments along [100] and [010] directions, respectively. The corresponding SAED pattern is displayed in **b**. **c**, schematic featuring edge dislocations, climbing of edge dislocations and screw part of mixed dislocations on (001) plane. Note that both dislocation climbing and screw part of mixed dislocations could be imaged in short dislocation segments along either [100] or [010] direction when viewed on (001) plane. The length of the viewed screw part may be closely related to TEM sample thickness, while the length of the dislocation climb segment was reported to be around 50-100 nm[1].

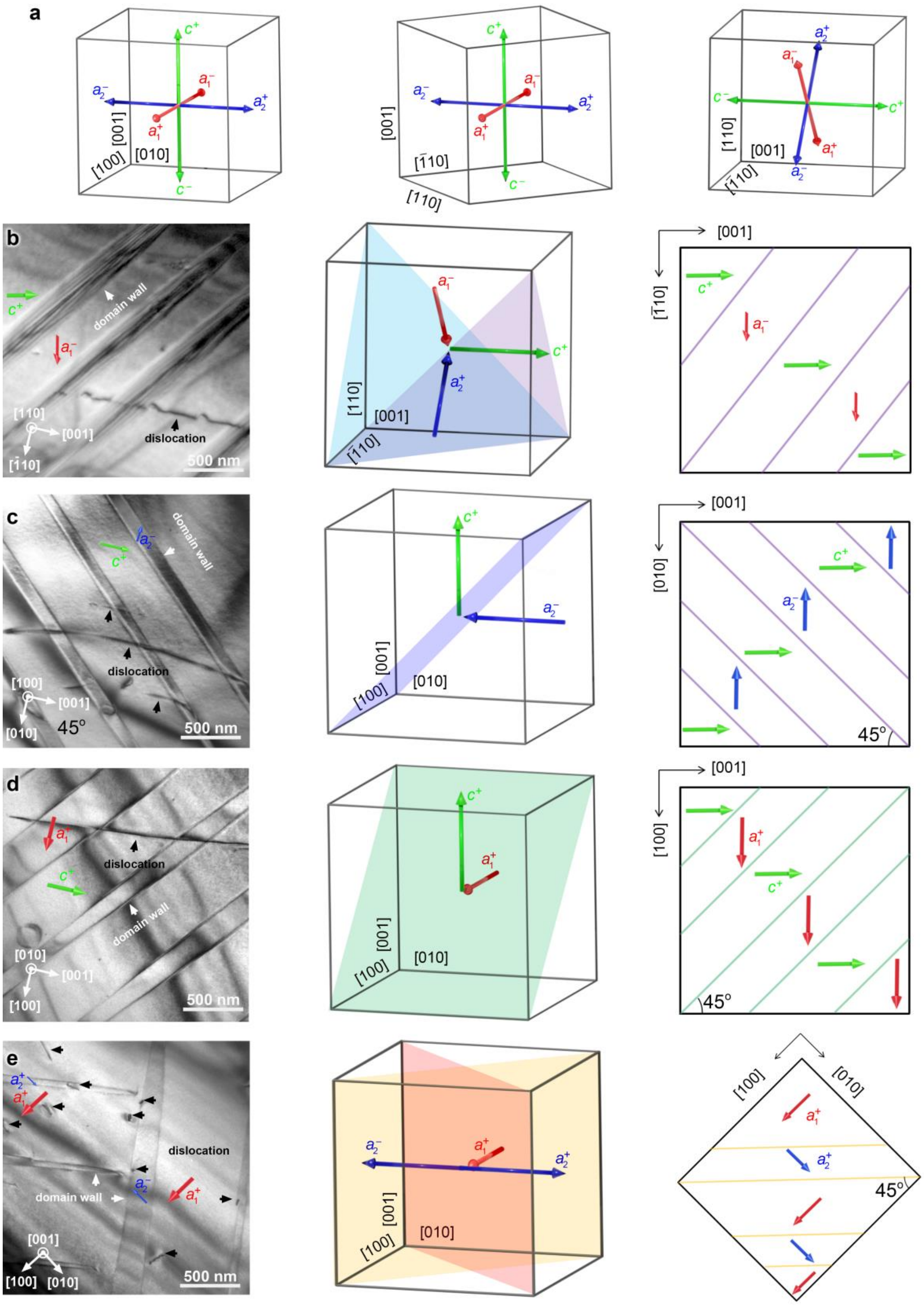

Supplementary Figure 6. Formation of 90° domain walls in the deformed tetragonal $BaTiO_3$ single crystals at room temperature. a, Schematics of the six polarization vectors in a tetragonal system with three different coordinate systems. Red, blue and green arrows indicate different polarization vectors parallel to [100] (defined as a_1 domain variants), [010] (that is, a_2 domain variants) and [001] (c domain variants), respectively. When looking from the [110] direction, asymmetrical fringes from a_1–c type 90° domain wall variants are very pronounced, as revealed in **b**. When a pair of

the {101} domain boundaries gets too close to each other, fringe patterns develop to become effectively symmetric ones along the center line of the domain[2]. The domain walls viewed edge-on are the 90° *a*–*c* type with the respective polarization vector being perpendicular to each other, see a_2–c 90° domain wall variants in **b**, and a_1–c 90° domain wall variants in **c**. The diagnostic feature for identifying the a_1–a_2 90° domain variants is the domain boundary of [110] or $[\bar{1}10]$ appearing edge-on, as schematically highlighted in **d**, **e**. We observed a_1–a_2 90° domain walls with the domain boundaries of both [110] (a_1^+–a_2^- variants) and $[\bar{1}10]$ (a_1^+–a_2^+ variants). Definition of a_1, a_2 and *c* domain variants can be found in Supplementary Note 1.

Sample	Small-signal permittivity at RT	T_C (°C)	P_{max} (μC cm^{-2})	P_r (μC cm^{-2})	E_c[a] (kV cm^{-1})	E_i[b] (kV cm^{-1})	Ref.
Reference (001)-cut	~2000	133	26.0	-	-	0	3
Reference (110)-cut	2093	-	20.6	18.4	0.53	0	4,5
Reference (001)-cut	1960	133	27.3	25.6	0.98	0	This work
Reference (110)-cut	1322	133	21.4	17.8	1.15	0	This work
Deformed (001)-cut	2901	134	13.3	8.31	3.03	0.65	This work
Deformed (110)-cut	1793	135	13.9	8.23	1.58	0.10	This work

Supplementary Table 1. Comparison of small-signal and large-signal properties of reference and deformed single-crystal $BaTiO_3$ samples. Note that small-signal permittivity at room temperature was determined at 1 kHz. Large-signal polarization and E_c in Ref.[3,5] and this work were obtained at 1 Hz, and these values in Ref.[4] were recorded at 10 mHz. [a] $E_c = |E_c^+ - E_c^-|/2$ and [b] $E_i = |E_c^+ + E_c^-|/2$, where E_c and E_i are the coercive field and internal bias field, respectively.

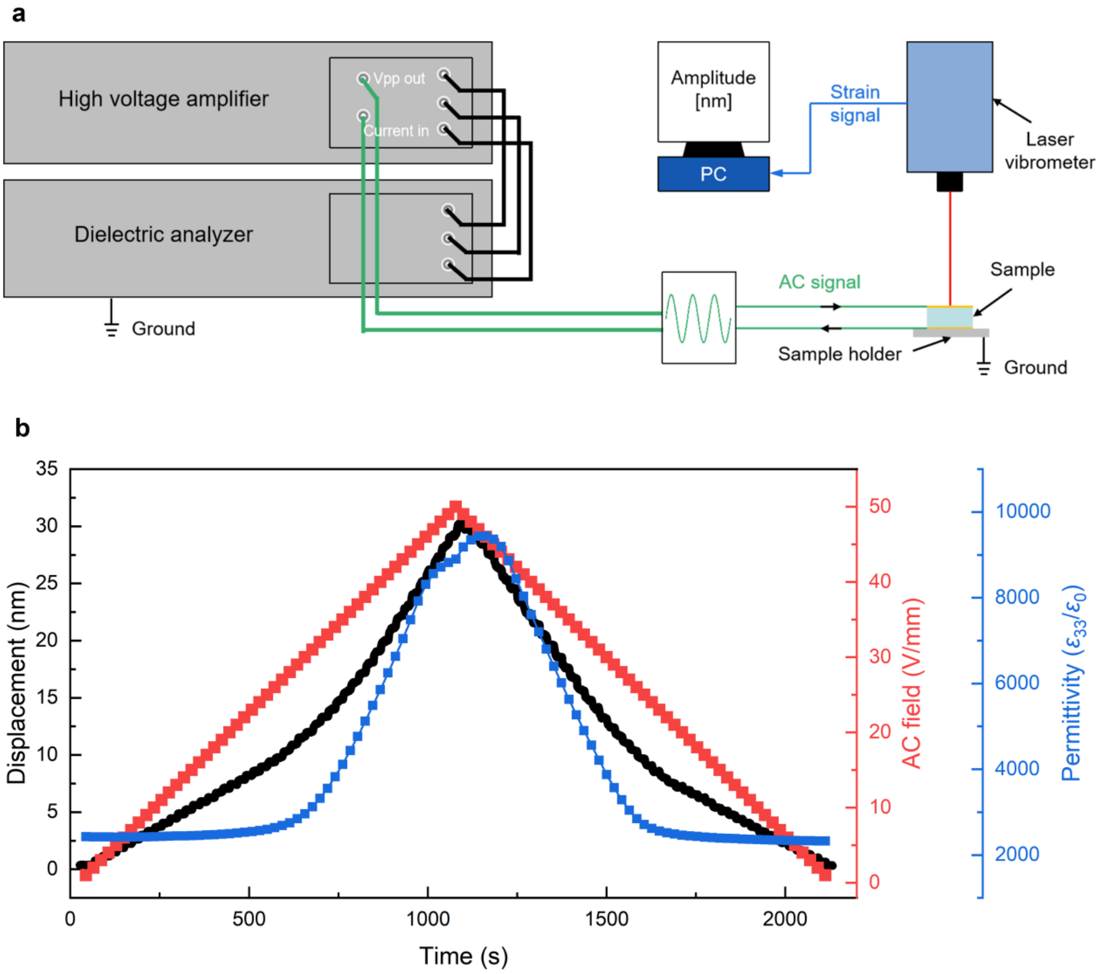

Supplementary Figure 7. Setup for permittivity and d_{33}* measurements. a, The poled sample was placed in the center of the sample holder. Input sinusoidal AC voltage signals were generated by a high voltage amplifier at a frequency of 1 kHz. However, the real resolution of the laser vibrometer is limited by the background noise (10–20 pm). Permittivity, amplitude of AC field, and displacement as a function of time can be recorded simultaneously using this setup. As one example, we used a test sample and plotted the data in **b**. Therefore, the corresponding converse piezoelectric coefficient can be calculated using the following equation, d_{33}* = displacement/amplitude of AC field.

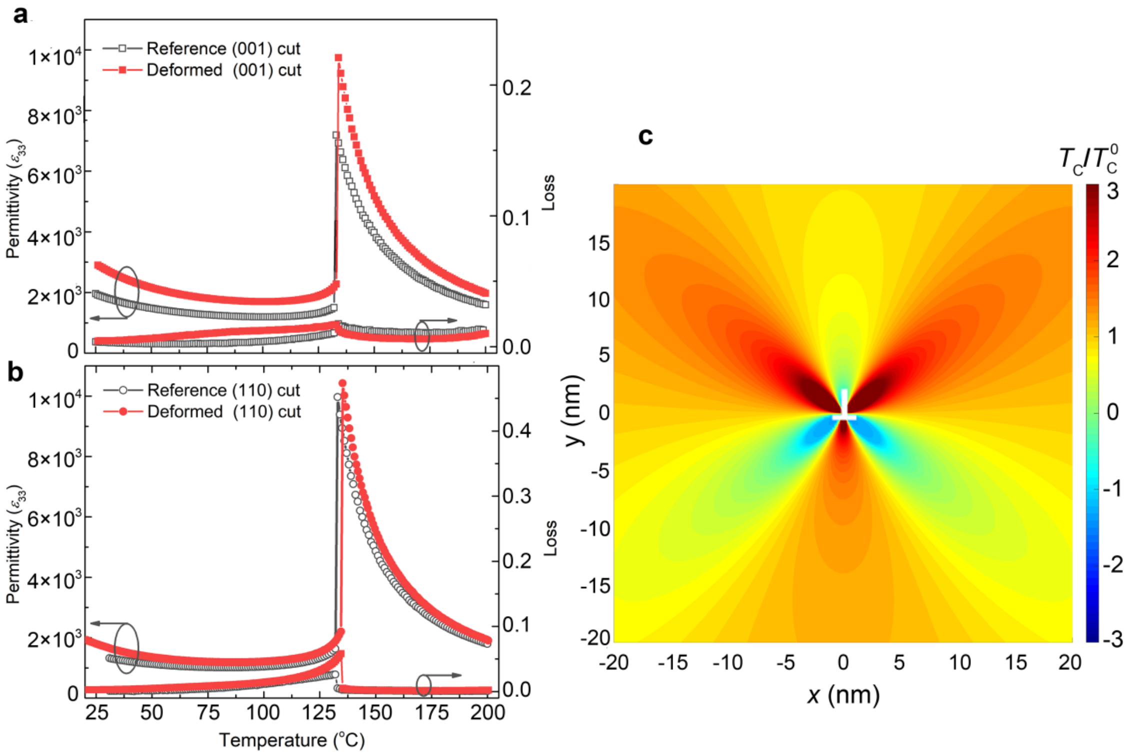

Supplementary Figure 8. Influence of dislocations on small-signal dielectric properties. Temperature dependence of small-signal dielectric permittivity and loss at 1 kHz for **a**, reference and deformed (001)-cut samples, and **b**, reference and deformed (110)-cut samples. The Curie point (T_C) of deformed samples was slightly enhanced as compared to reference sample. **c**, Normalized T_C, $T_C/T_C^0(x,y)$ map near an edge dislocation core, where T_C^0 is the Curie point without dislocation. The local enhancement of T_C is correlated with the appearance of mechanically imprinted dislocation structures due to the local strain field generated by a single dislocation with Burgers vector along [010] and magnitude $|\vec{b}| = a$, where a = 0.396 nm is the lattice constant[6]. Approaching the dislocation core, strain field is so strong that ferroelectricity sets in. Farther away from the dislocation core, the strain field is weak so that T_C enhancement is small[3].

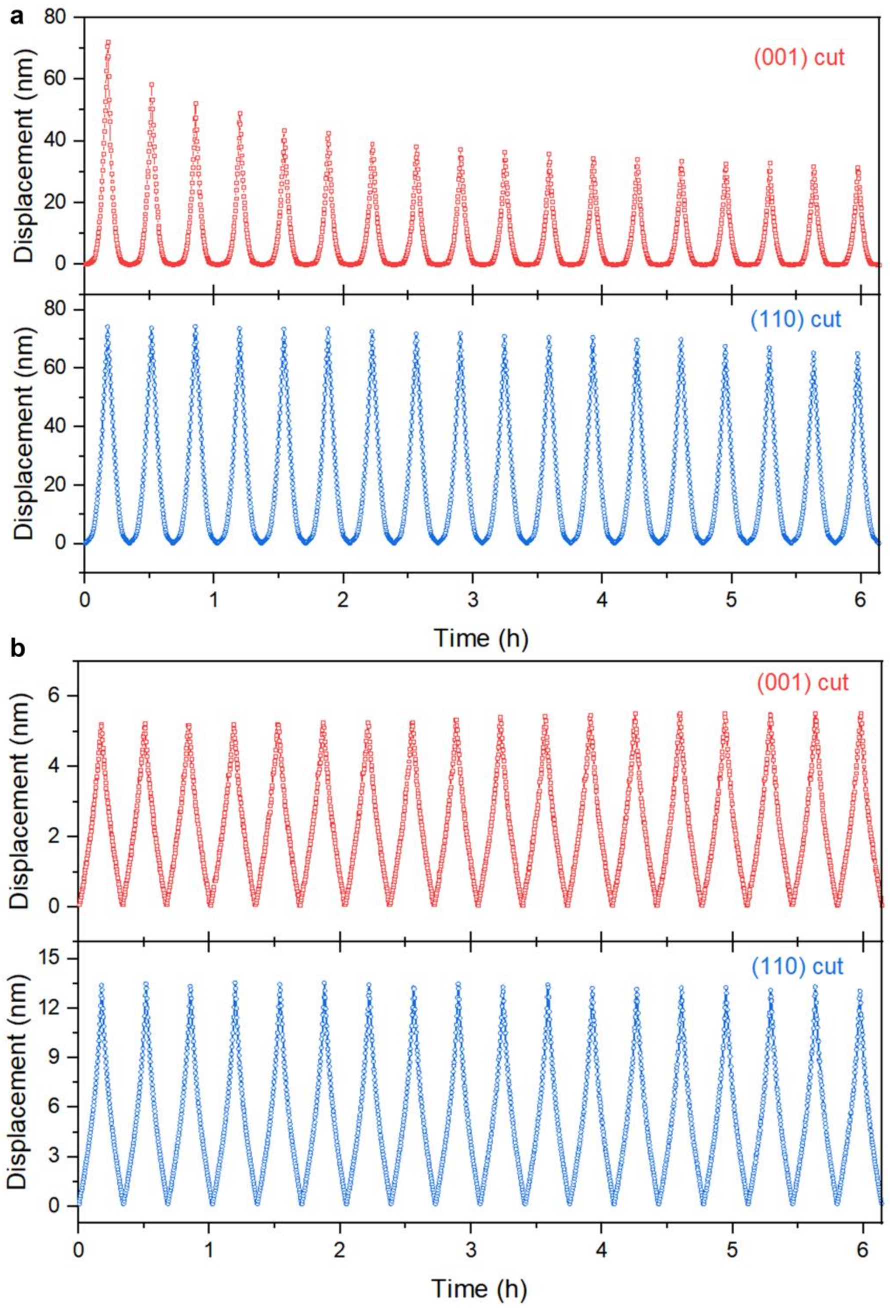

Supplementary Figure 9. Displacement as a function of time. a, Displacement of (001)-cut and (110)-cut deformed samples. **b**, Displacement of (001)-cut and (110)-cut reference samples.

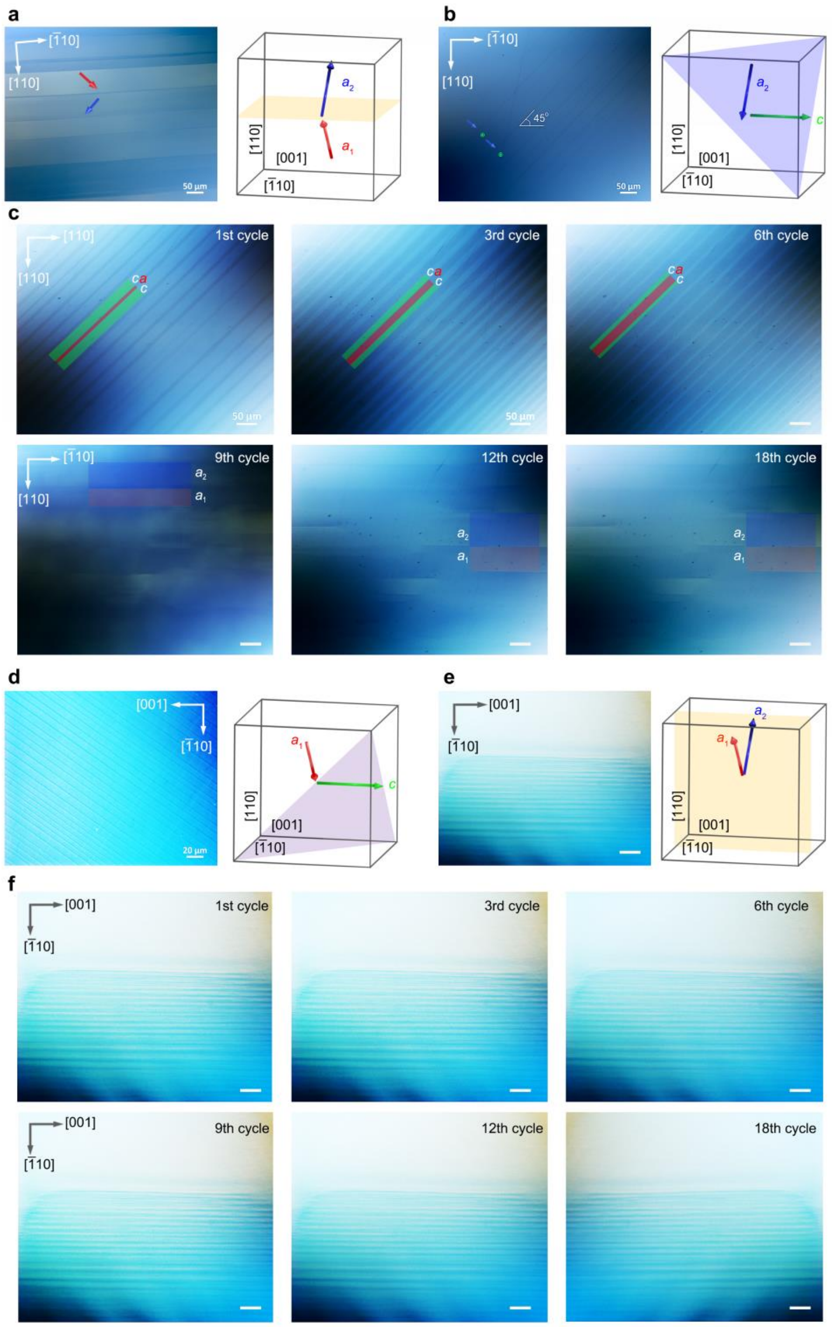

Supplementary Figure 10. Observation of domain structures using optical microscopy. (001)-cut deformed sample has a_1–a_2 90° domain walls in unpoled state, see optical image and schematic in **a**. After poling, a_2–c 90° domain walls were observed, as displayed in **b**. Obvious changes in domain patterns were imaged when experiencing one cycle (1→50→1 V mm^{-1}), as displayed in **c**. Scale bar: 50 μm. a_2–c 90° domain walls transformed into a_1–a_2 90° domain walls during the cycling process. Domain variants shown in **c** are marked by different colors. (110)-cut deformed sample has a_1–c 90° domain walls in unpoled state and a_1–a_2 90° domain walls in poled state, as depicted in **d** and **e**, respectively. The a_1–a_2 90° domain walls remained stable during the AC field cycling (see **f**). Scale bar: 20 μm.

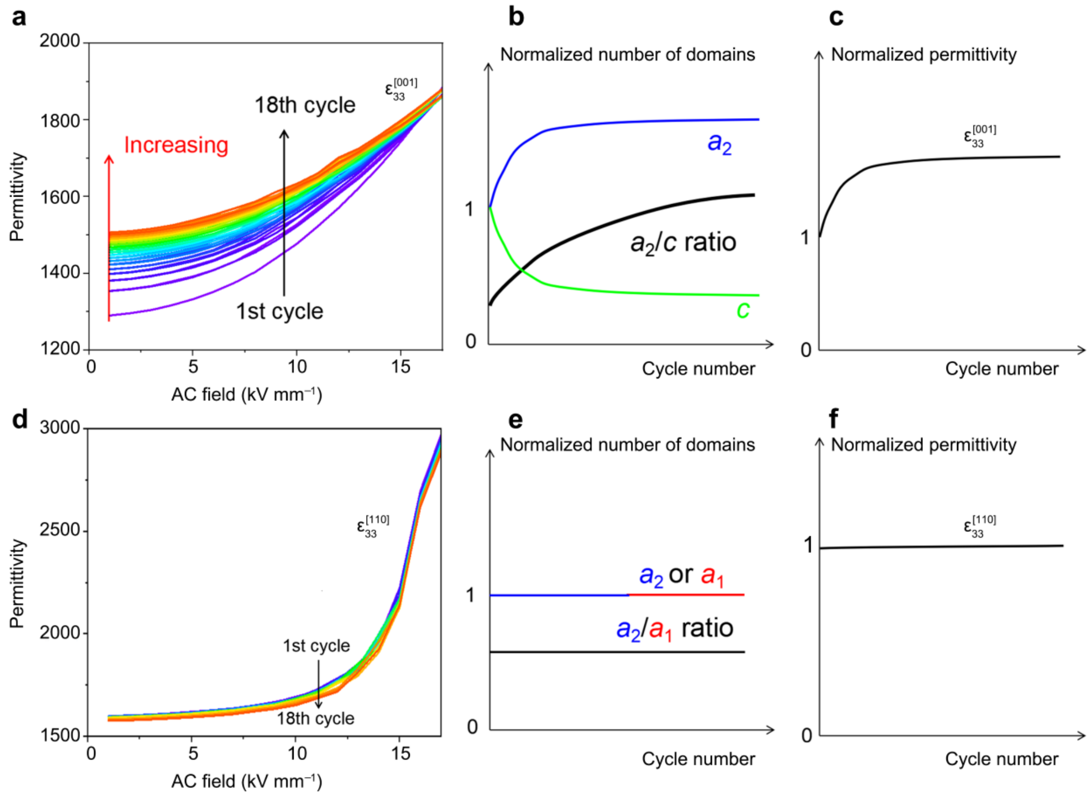

Supplementary Figure 11. Anisotropic stability of dielectric permittivity and domains during cycling. **a**, Dielectric permittivity and **b**, a_2/c domain ratio of (001)-cut deformed sample increased with increasing cycle number. **c**, The increase in the permittivity of (001)-cut sample can be rationalized due to the anisotropy of the dielectric tensor of $BaTiO_3$ single crystal, with $\varepsilon_a > \varepsilon_c$. **d**, Dielectric permittivity and **e**, a_2/a_1 domain ratio of (110)-cut deformed sample remained nearly the same with increasing cycle number, leading to a stable permittivity at low fields in **f**. The normalized permittivity was divided by the initial permittivity value of the first cycle.

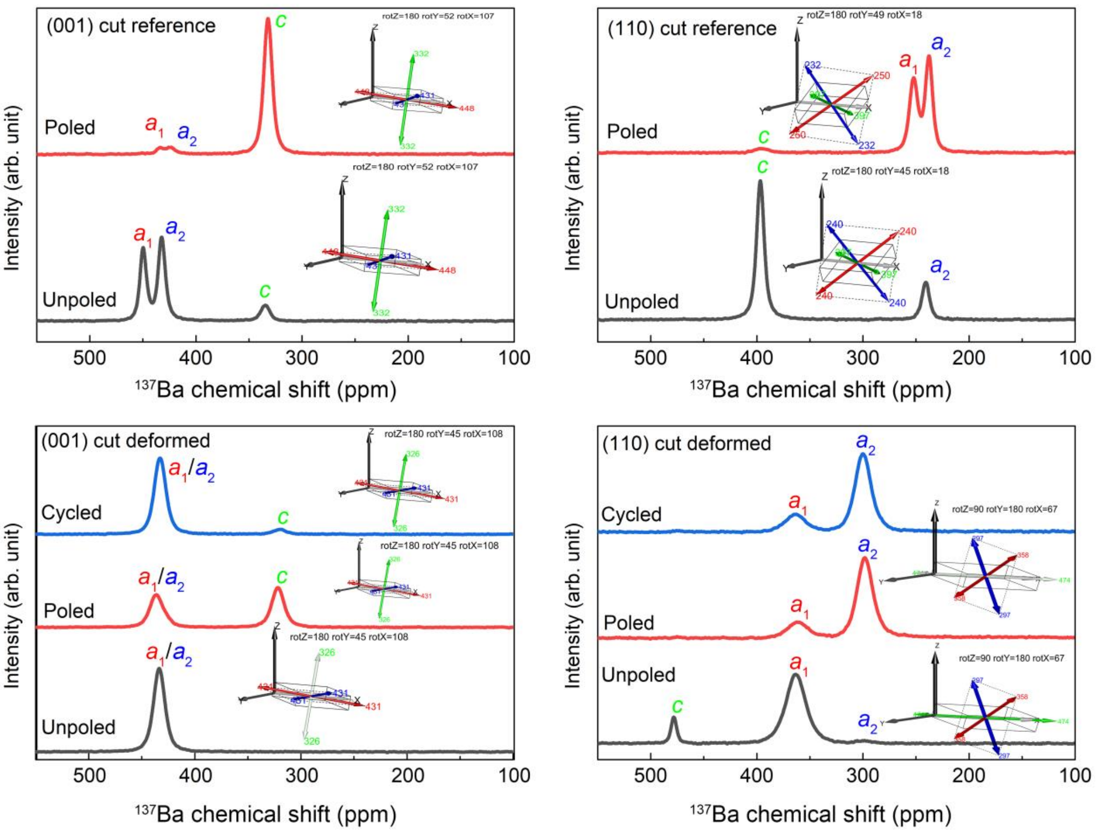

Supplementary Figure 12. Determination of domain configurations of both (110)- and (001)-cuts in unpoled, poled and cycled states by ^{137}Ba nuclear magnetic resonance (NMR) spectroscopy. Green color indicates *c*-domains with polarization vector pointing toward [001], red and blue colors signify a_1-domains (polarization vector pointing toward [100]) and a_2-domains (polarization vector pointing toward [010]). The rotation axis (X-axis provided in the insets) is perpendicular to the applied magnetic field B_0 (Z-axis). Based on the crystal orientation and the constraint of orthogonal domain orientation for $BaTiO_3$ we can assign particular signal positions to specific domain orientations by exploring the orientation-dependence of the second-order quadrupolar interaction[7]. The signals' areas are used to determine the relative amounts of individual domain configurations in our samples. After poling along the [001] direction, the (001)-cut reference sample has more *c*-domains, but the formation of *a*–*c* 90° domain wall variants is preferred due to the strain field surrounding the dislocations. After poling along the [110] direction, both reference and deformed (110)-cut samples favored a_1–a_2 90° domain wall variants. An angle of 20° was manually set to measure samples exposing a (001) face or a (110) face, respectively. We corrected this angle by performing the calculation of the positions of each NMR spectroscopy. It seems that the actual angle is 17° or 18° (namely, 2° or 3° offset) for the NMR experiments, a discrepancy that may arise both from the specific crystal cut and uncertainty in orientation of single crystals on the goniometer tenon.

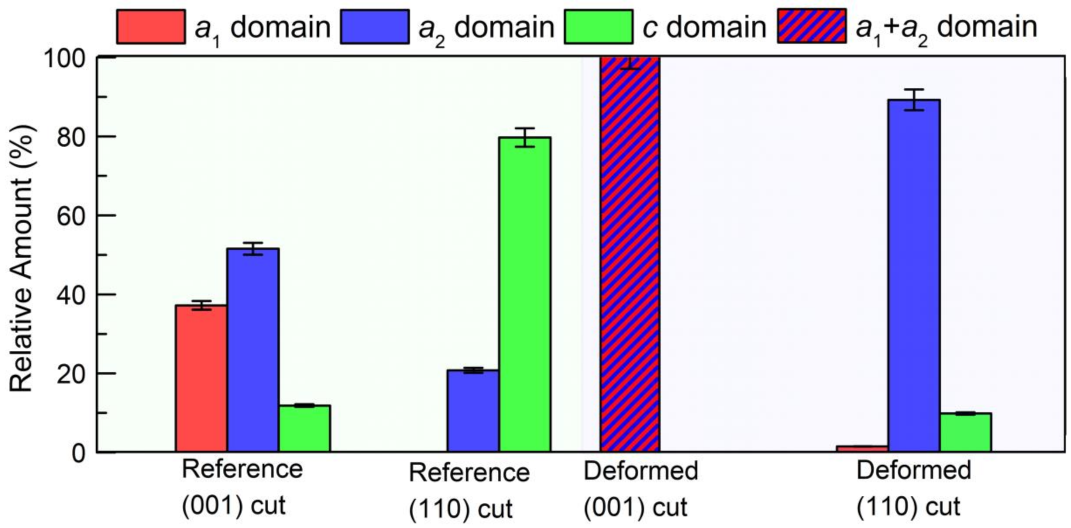

Supplementary Figure 13. Results of the domain fraction analysis by ^{137}Ba NMR spectroscopy. Relative amounts in (001)- and (110)-cut samples in unpoled state.

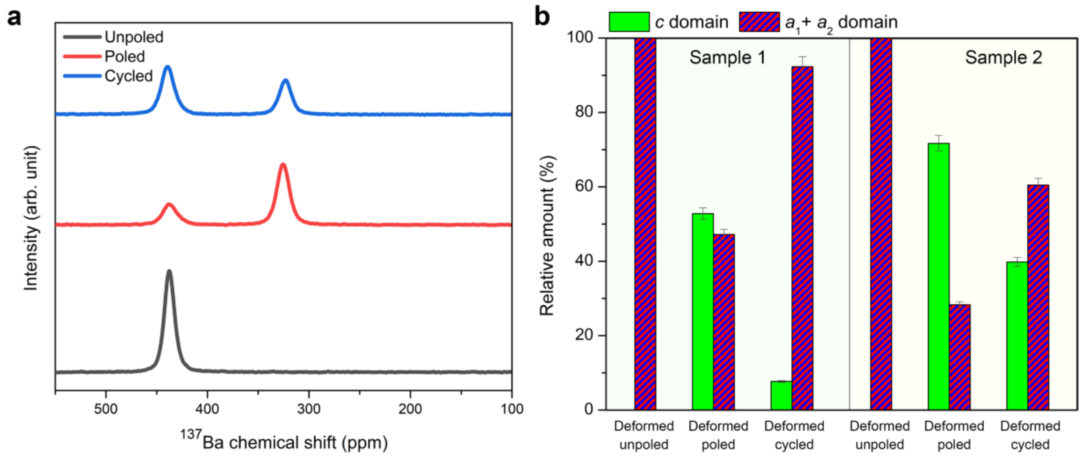

Supplementary Figure 14. Time dependence of relative amount in (001)-cut deformed samples. Additional NMR experiments were carried out to investigate the influence of waiting time between poling and NMR experiment. The NMR data of sample 1 were used in the main text (see Fig. 3d) because the experimental conditions kept the same as we had used for electrical measurements. For sample 1, the waiting times after poling for poled and cycled samples were about 11 h and 20 h, respectively. For sample 2, the waiting times after poling for poled and cycled samples were about 2 h and 4 h, respectively. Sample 2 had a shorter waiting time as compared to sample 1. Therefore, we obtained the same trend in unpoled, poled and cycled states (see **a**), but with more *c*-domains in poled and cycled states when compared to that of sample 1 (see **b**).

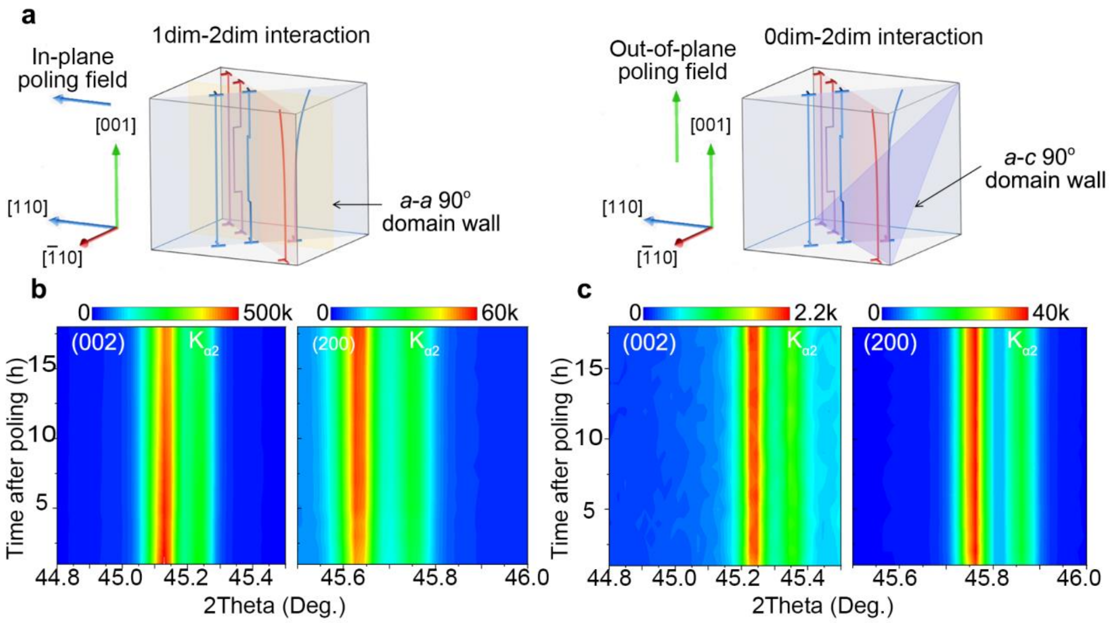

Supplementary Figure 15. Texture analysis of (200) and (002) reflections using XRD. a, Schematics depicting in-plane and out-of-plane poling to get 1-dim/2-dim and 0-dim/2-dim interactions for XRD experiments. The intensity of (002) reflection decreased and that of (200) reflection increased with increasing time after out-of-plane poling, while these intensities were stable for in-plane poling case, as verified in **b** and **c**. Therefore, the *a*/*c* ratio of the out-of-plane poling (namely, 0-dim/2-dim interaction) increased with increasing time after poling, as summarized in Fig. 3**e** in the main text. Note that the *a*/*c* ratio of 0-dim/2-dim interaction is not stable without AC field cycling.

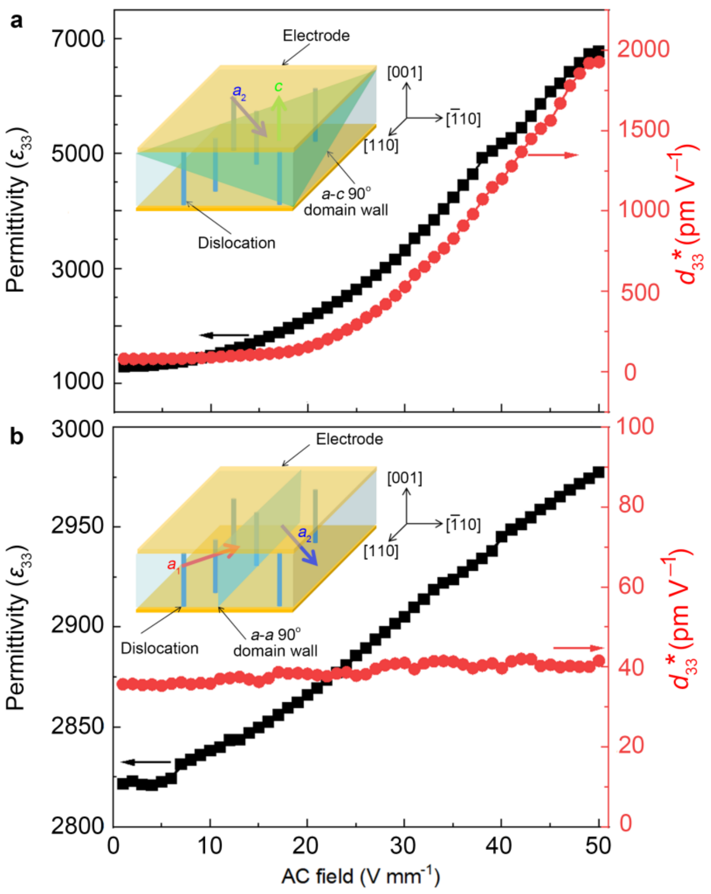

Supplementary Figure 16. Dielectric and electromechanical response of the (001)-cut deformed sample with *a*–*c* and *a*–*a* domain configurations. We applied the AC field along the [001] direction and measured the permittivity and d_{33}* simultaneously. **a**, the driving force can move the a_2–c 90° domain wall through 0-dim/2-dim interaction (as shown in the inset), leading to large permittivity and d_{33}*. However, there is no driving force for the motion of a_1–a_2 90° domain wall even if the domain wall is parallel to dislocations via 1-dim/2-dim interaction, as highlighted in the inset in **b**. As a result, domain-wall pinning cannot contribute to the dielectric permittivity and d_{33}* since there is no driving force for domain-wall motion (see **b**). We therefore conclude that the driving force and dislocation-domain wall configuration are crucial for designing dislocation-based functionalities.

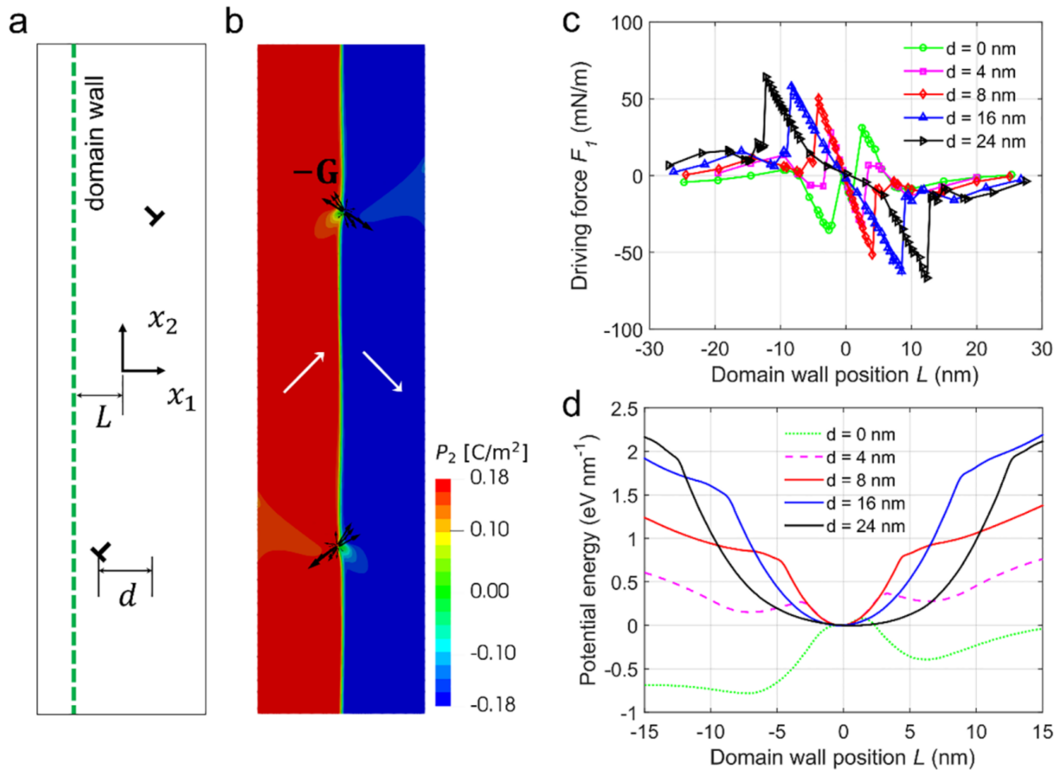

Supplementary Figure 17. Phase-field simulations of the dislocation-induced driving force on the domain wall and the corresponding potential energy landscape. a, Schematic of the numerical model with the size of 100×400 nm^2. The dislocation spacing was set as 200 nm. The distance between dislocations is d in x_1 direction. **b**, Interaction between dislocations and domain wall, and the distribution of nodal configurational force –**G** around the dislocations. d = 4 nm is simulated. **c**, Driving force on the domain wall induced by dislocations for different d. **d**, Potential energy landscape for different d.

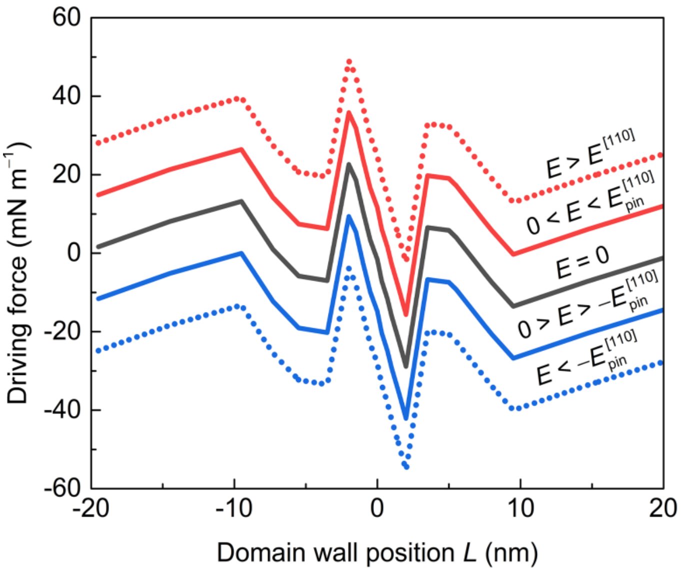

Supplementary Figure 18. Phase-field simulations of the driving force on the domain wall with *d* = 4 nm under different electric fields. Simulated driving force as a function of domain wall position for 1-dim/2-dim DDW interactions. The corresponding potential energies of 1-dim/2-dim interaction are plotted in Fig. 4g.

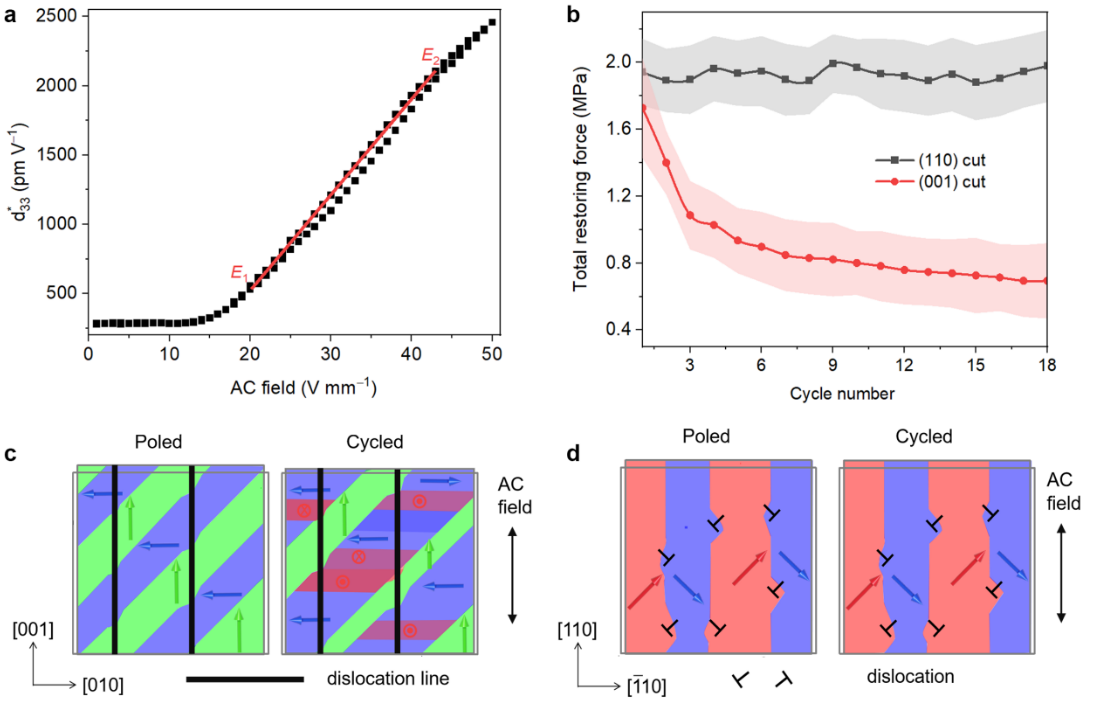

Supplementary Figure 19. Comparison between macroscopic restoring force and local pinning force. a, A representative d_{33}* *vs* field curve with the linear regime (marked between E_1 and E_2) was used to calculate the macroscopic restoring force. **b**, The plots of calculated macroscopic restoring forces for both (001)- and (110)-cut deformed samples. Schematic depictions of the changes of domain structures in **c**, (001)-cut deformed sample and **d**, (110)-cut deformed sample during AC field cycling.

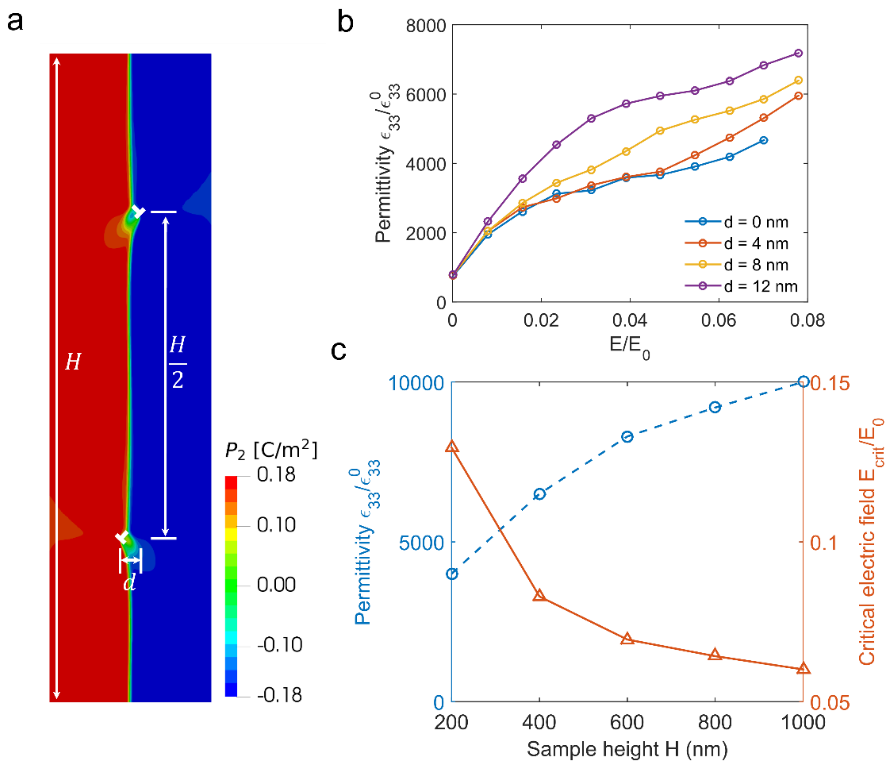

Supplementary Figure 20. Computed dielectric permittivity for the the (110)-cut deformed sample by considering two dislocation lines and a 90° domain wall. a, Numerical model, where the dislocations are separated by a distance of H/2. **b**, Dependence of permittivity on the electric field. **c**, Critical electric field (pinning field) and the corresponding permittivity with respect to sample height. E_0 = 9.65 kV cm^{-1}. According to equations (9) and (10) in the main text, the pinning force is proportional to the pinning field, but the force is proportional to the number of dislocations per area. Therefore, increasing the sample height corresponds to a reduction of the dislocation density. With increasing dislocation density, the pinning field increases while the permittivity decreases. It should be noted that both 90° and 180° walls will contribute to the permittivity. The experimentally observed dielectric permittivity ε_{33} cannot be quantitatively simulated without knowledge of the complex 3D dislocation-domain wall interaction, as summarized in Supplementary Figure 21.

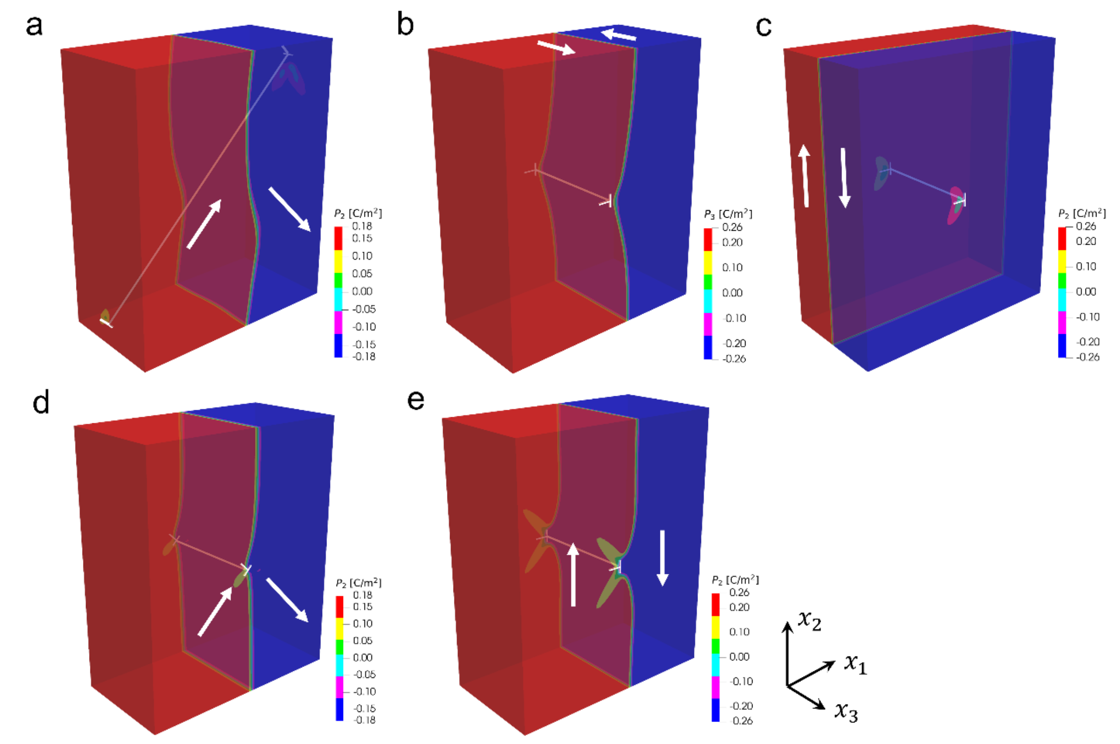

Supplementary Figure 21. Dislocation-domain wall configurations. The sample size is 100×120×50 nm^3. **a**, Intersection model for dislocation and 90° domain wall in both (110) and (001)-cut samples. **b**, Parallel model for dislocation and 180° domain wall in the (001)-cut sample. **c**, Intersection model for dislocation and 180° domain wall in the (110)-cut sample. **d**, Parallel model for dislocation and 90° domain wall in the (110)-cut sample. **e**, Parallel model for dislocation and 180° domain wall in the (110)-cut sample. The external electric field is applied in the x_3 direction for **b**, and in the x_2 direction for all other configurations.

Fig. S21	DW type	Burgers vector	Analytical force on dislocation (N m^{-1})	Numerical E_{pin} (kV mm^{-1})	Numerical pinning force (nN)	Pinning stress (MPa)
a	90	$b_0[00\bar{1}]$	$\nu\sigma_0 b_0/\sqrt{2}$	0.08	0.18	0.024
		$b_0[\bar{1}10]/\sqrt{2}$	0	0	0	0
b	180	$b_0[100]$	0	0.26	0.81	0.108
		$b_0[010]$	$\nu\sigma_0 b_0$	0.27	0.84	0.112
c	180	$b_0[100]$	0	0.04	0.12	0.016
		$b_0[010]$	0	0	0	0
d	90	$b_0[110]/\sqrt{2}$	$\sigma_0 b_0/\sqrt{2}$	1.87	4.12	0.550
		$b_0[1\bar{1}0]/\sqrt{2}$	$-\sigma_0 b_0/\sqrt{2}$	–1.87	–4.12	–0.550
e	180	$b_0[100]$	0	0.37	0.82	0.109
		$b_0[010]$	$\sigma_0 b_0$	3.77	8.32	1.10

Supplementary Table 2. Pinning force for all possible DDW configurations. In general, both 180° and 90° domain walls are allowed in a polar tetragonal symmetry. The magnitude of the Burgers vector is b_0 and perpendicular Burgers vectors are considered for each model. The stress field of the domain wall in the x_i coordinate system is $\sigma_{22} = \sigma_0$ and $\sigma_{33} = \nu\sigma_{22}$, where ν is the Poisson's ratio. The analytical force on the dislocation line (force per unit length) is calculated using Peach-Koehler force formula[8] and only the component perpendicular to the domain wall plane is shown in the table. The analytical solution helps a qualitative determination of the pinning force induced by the dislocation. The numerical results show that the pinning force is strongly influenced by the nucleation of domains due to stress field of the dislocation. The numerical pinning force is calculated as $F = 2P_2E_{\mathrm{pin}}A$, where E_{pin} is the numerical critical electric field and $A = 120\times50$ nm^2 is the area of the cross-section of the sample. Strong pinning forces can only be observed in the (110)-cut sample with the configurations in Fig. S21 (a,c,d,e) for both 180° and 90° domain walls.

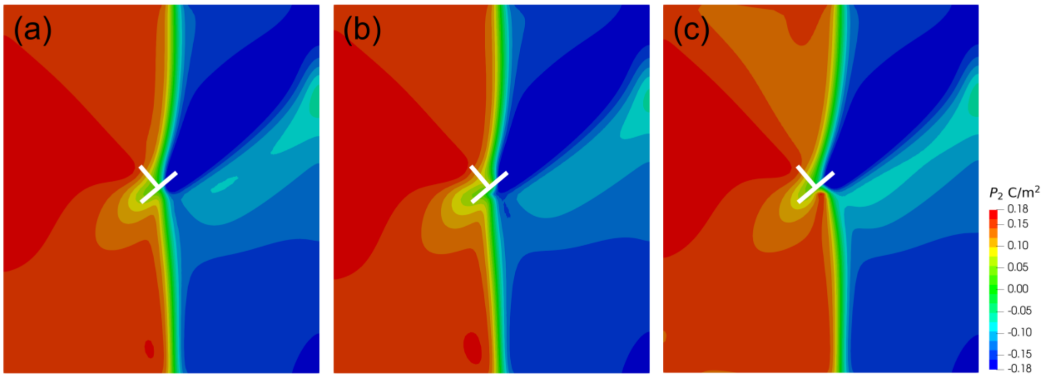

Supplementary Figure 22. The influence of charges on domain-wall pinning. The sample size is 60×70 nm^2. **a**, uncharged dislocation at critical pinning electric field of 2.21 kV mm^{-1}. **b**, Dislocation with positive charge at critical pinning electric field of 2.20 kV mm^{-1}. **c**, Dislocation with negative charge at critical pinning electric field of 2.12 kV mm^{-1}. The point charge is set to be $Q = P_s l_0$, where $P_s = 0.26$ C m^{-2} is the spontaneous polarization of $BaTiO_3$ and l_0 is 1 nm. Therefore, the influence of charge is relatively small.

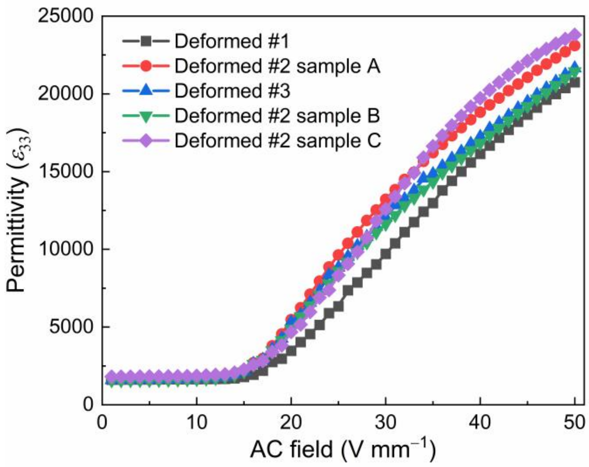

Supplementary Figure 23. Effect of dislocation density on properties over different deformations with the same loading parameters. AC field dependence of permittivity for the (110) cut samples obtained from three deformations with a loading rate of 0.2 N s^{-1} at 1150 °C. We cut three samples (A, B and C) from the different parts of the deformed #2 crystal to investigate the effect of the distribution of dislocation density on properties. Permittivity data were measured at 1 kHz.

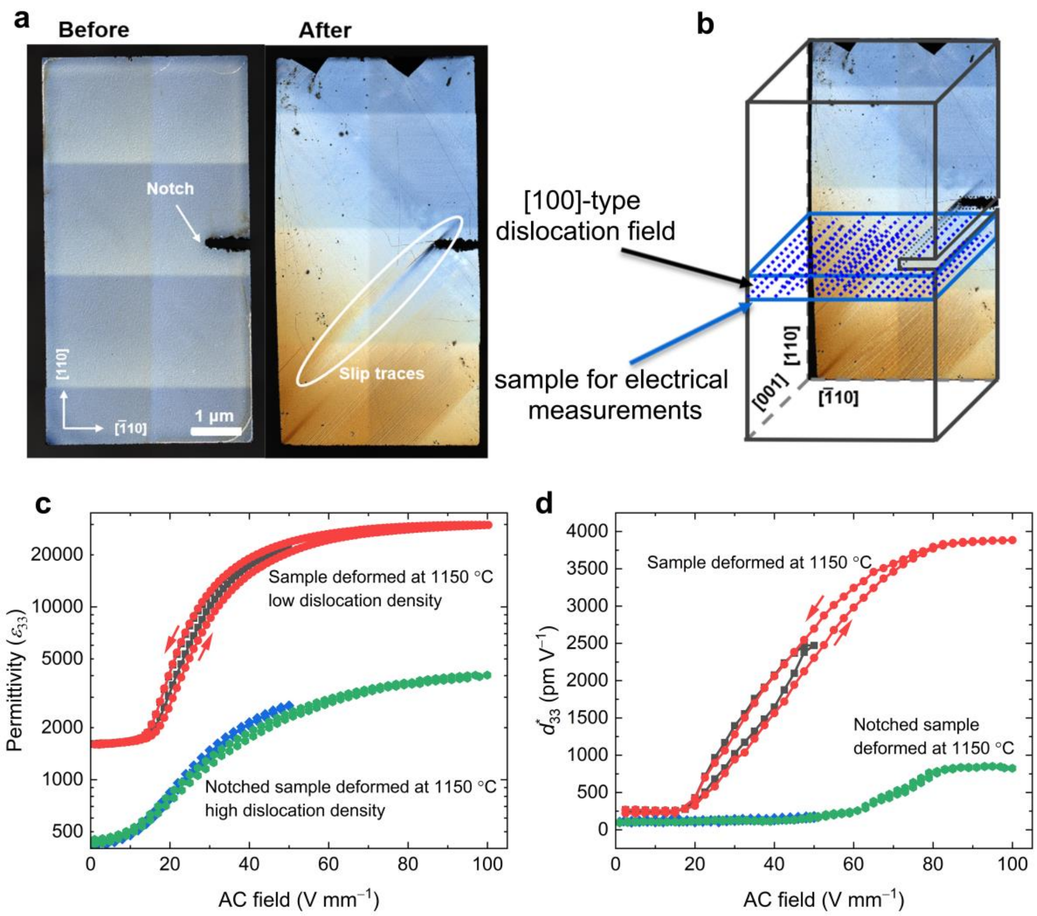

Supplementary Figure 24. Effect of dislocation density on dielectric and piezoelectric properties. a, **b**, Deformation of $BaTiO_3$ single crystal with a notch leads to the emission of dense and ordered [100]-type dislocations from the notch, and {100}<100> dislocation fields are formed. Clear slip traces were observed on the (001) planes. **c**, **d**, Comparison of AC field dependence of permittivity and d_{33}* for (110) cut samples with and without a notch. Electrical properties were measured at 1 kHz.

Supplementary Note 1.

Definition of *a* and *c* domains in a tetragonal phase

For a tetragonal $BaTiO_3$, three different crystallographic domains (a_1, a_2, and c) can be defined based on the directions of six spontaneous polarization vectors[9]. To avoid any confusion in the definition of a_1-, a_2-, and c-domains of both (001)- and (110)-cut samples, we highlight the relative orientation of these three domains as follows: a_1-, a_2-, and c-domains refer to the (±)-orientation of the spontaneous polarization with respect to the [100], [010], and [001] directions, respectively. Specifically, (001)-cut samples have ferroelectric domains whose polarization points along either of the two in-plane crystallographic directions are termed a_1- and a_2-domains whereas out-of-plane domains are termed c-domains, as defined in the middle of Supplementary Figure 6a. For (110)-cut samples, c-domains are oriented in the in-plane crystallographic directions [001] (c^+-domain) or $[00\bar{1}]$ (c^--domain), while a_1^+–a_2^+ and a_1^-–a_2^- domain variants have 45° with the crystallographic [110] direction (see the right schematic in Supplementary Figure 6a), respectively. In the whole paper, red, blue and green arrows indicate a_1-, a_2- and c-domain variants, respectively.

Supplementary Note 2.

Comparison between macroscopic restoring force and local pinning force

In the main text, the configurational force theory is employed to calculate the dislocation-induced driving force on the domain wall as well as the external electric field-induced driving force. In general, the total restoring force (F_r) consists of both mechanical restoring force (F_{mr}) associated with an increase in the elastic energy that is caused by domain wall displacements and dislocation-related restoring force (F_{dr}) on the domain wall motion[10,11]:

$$F_r = F_{mr} + F_{dr} \tag{S1}$$

For a 90° domain wall at low frequencies (< 100 kHz), the mass of the domain wall can be neglected and the restoring force (F_r) is written in terms of a domain wall stiffness k defined as

$$F_r = -kl \tag{S2}$$

where l is the amplitude of the field-induced domain wall oscillations. Macroscopically, we are able to account the total strain (S) in the intermediate electric field regime by considering the linear back-stress upon reduction of electric field between the low field E_1 and high field E_2 (see Supplementary Fig. 19a)

$$S = \int_{E_1}^{E_2} d_{33}^{*}(E) \cdot dE \tag{S3}$$

Then, the total macroscopic restoring force during the linear back-stress upon reduction of electric field is written as

$$F_r = \frac{Y}{1-\nu^2} \times S \tag{S4}$$

where Y is the Young's modulus and ν is the Poisson ratio, respectively. We take Y = 63.6 GPa for [001]-oriented crystal and Y = 54.3 GPa for [110]-oriented crystal[12] with a Poisson ratio of ν = 0.3. The calculated macroscopic total restoring forces, using experimental results for d_{33}* and Eqs. (S3) and (S4), for both (001)- and (110)-cut

deformed samples are plotted in Supplementary Fig. 21b. Our simulated local pinning stresses are 0.43 MPa and 0.029 MPa for (110)-cut sample and (001)-cut sample, respectively. The local pinning stresses are smaller than the measured values in Supplementary Fig. 19b, revealing that the mechanical restoring force caused by strain incompatibility plays an important in the back-switching[4].

We observed that the *c*/*a* domain ratio of (001)-cut deformed sample increased during the cycling, as schematically shown in Supplementary Fig. 19c. The corresponding change in the out-of-plane permittivity (ε_{33}) can be evaluated as[13]

$$\varepsilon_{33} = \frac{d < P_3 >}{dE} = \phi_c \frac{dP_3}{dE} + P_3 \frac{\phi_c}{dE} \tag{S5}$$

where ϕ_c is the fraction of the *c*-domains. The first term indicates the intrinsic permittivity of the polydomain state and the second term represents the extrinsic contribution to the permittivity arising from the reversible displacement of the domain walls that changes the domain fractions. By analogy with domain wall vibrations in ferroelectric polycrystalline ceramic system[10,13], the extrinsic domain wall contribution to the permittivity can be expressed as

$$\varepsilon = \frac{2\sqrt{2}P_s^2}{\varepsilon_0 kD} \tag{S6}$$

where P_s is the spontaneous polarization, ε_0 is the permittivity of the vacuum, and D is the average domain periodicity, respectively. The concept of domain wall stiffness has been invoked for many years[10,11,13,14] to understand the displacement of domain walls. In real samples, k is frequency dependent even in low frequency regime (1–100 kHz) due to domain wall pinning. It was experimentally reported that k increased with increasing the fraction of *a*-domains[13]. Therefore, we conclude that the extrinsic contribution to the permittivity from 90° domain walls decreased during cycling process because domain wall stiffness k increased. The increase in the fraction of *a*-domains contribute to a higher permittivity at low AC field due to anisotropy of the dielectric tensor of $BaTiO_3$ single crystal, with $\varepsilon_a > \varepsilon_c$, as shown in Supplementary Fig. 11a. There is no driving force for the motion of a_1-a_2 90° domain walls when we

measure the d_{33}* along the [001] direction, as highlighted in Supplementary Fig. 15. As a result, a degradation in d_{33}* of the (001)-cut sample can be well-explained. For (110)-cut deformed sample, the strong restoring force at microscopic and macroscopic levels ensured reversible back-switching with stable domain wall patterns, see Supplementary Fig. 19d. Consequently, both permittivity and d_{33}* are stable during cycling.

Supplementary References

1. Doukhan, N. & Doukhan, J. C. Dislocations in perovskites $BaTiO_3$ and $CaTiO_3$. *Phys. Chem. Minerals* **13**, 403–410 (1986).

2. Chou, J. F., Lin, M. H. & Lu, H. Y. Ferroelectric domains in pressureless-sintered barium titanate. *Acta Mater.* **48**, 3569–3579 (2000).

3. Höfling, M., et al. Control of polarization in bulk ferroelectrics by mechanical dislocation imprint. *Science* **372**, 961–964 (2021).

4. Hubmann, A. H., Li, S., Zhukov, S., Von Seggern, H. & Klein, A. Polarisation dependence of Schottky barrier heights at ferroelectric $BaTiO_3/RuO_2$ interfaces: influence of substrate orientation and quality. *J. Phys. D: App. Phys.* **49**, 295304 (2016).

5. Wada, S., Takeda, K., Muraishi, T., Kakemoto, H., Tsurumi, T. & Kimura, T. Domain wall engineering in lead-free piezoelectric grain-oriented ceramics. *Ferroelectrics* **373**, 11–21 (2008).

6. Von Hippel, A. Ferroelectricity, domain structure, and phase transitions of barium titanate. *Rev. Mod. Phys.* **22**, 221–237 (1950).

7. Liechti, O & Kind, R. NMR-NQR rotation patterns of single crystals with quadrupolar inhomogeneities. *J. Magn. Reson.* **85**, 480–491 (1989).

8. Zhou, X., Liu, Z. & Xu, B.-X. Influence of dislocations on domain walls in perovskite ferroelectrics: phase-field simulation and driving force calculation. *Int. J. Solids Struct.* **238**, 111391 (2022).

9. Nordlander, J., et al. Ferroelectric domain architecture and poling of $BaTiO_3$ on Si. *Phys. Rev. Mater.* **4**, 034406 (2020).

10. Pertsev, N. A., Arlt, G. & Zembilgotov, A. G. Prediction of a giant dielectric anomaly in ultrathin polydomain ferroelectric epitaxial films. *Phys. Rev. Lett.* **76**, 1164–1367 (1996).

11. Pertsev, N. A. & Arlt, G. Forced translational vibrations of 90° domain walls and the dielectric dispersion in ferroelectric ceramics. *J. Appl. Phys.* **74**, 4105–4112 (1993).

12. Berlincourt, D. & Jaffe, H. Elastic and piezoelectric coefficients of single-crystal barium titanate. *Phys. Rev.* **111**, 143–148 (1958).

13. Karthik, J., Damodaran, A. R. & Martin, L. M. Effect of 90° domain walls on the low-field permittivity of $PbZr_{0.2}Ti_{0.8}O_3$ thin films. *Phys. Rev. Lett.* **108**, 167601 (2012).

14. Arlt, G. & Dederichs, H. Complex elastic, dielectric and piezoelectric constants by domain wall damping in ferroelectric ceramics. *Ferroelectrics* **29**, 47–50 (1980).
