# Supplementary material for: Anisotropic dislocation-domain wall interactions in ferroelectrics
Source: Nat Commun. 2022 Nov 5;13:6676. doi: 10.1038/s41467-022-34304-7 (PMC9637100; doi:10.1038/s41467-022-34304-7)
Supplement: Supplementary file 2 — Description of Additional Supplementary Files [file 41467_2022_34304_MOESM2_ESM.pdf]

Description of Additional Supplementary Files

Supplementary Movie 1. Interactions between dislocations and a 90° domain wall for (001)-cut sample.

Supplementary Movie 2. Interactions between dislocations and a 90° domain wall for (110)-cut sample
